# Retrospective and Prospective Look at Aflatoxin Research and Development from a Practical Standpoint

**DOI:** 10.3390/ijerph16193633

**Published:** 2019-09-27

**Authors:** Noreddine Benkerroum

**Affiliations:** Department of Food Science and Agricultural Chemistry, Macdonald-Stewart Building, McGill University, Macdonald Campus, 21,111 Lakeshore Road, Sainte-Anne-de-Bellevue, QC H9X 3V9, Canada; n.benkerroum@gmail.com; Tel.: +1-514-652-4945

**Keywords:** aflatoxins, liver cancer, public health risk, foods and feeds, control means, climatic change

## Abstract

Among the array of structurally and toxicologically diverse mycotoxins, aflatoxins have attracted the most interest of scientific research due to their high toxicity and incidence in foods and feeds. Despite the undeniable progress made in various aspects related to aflatoxins, the ultimate goal consisting of reducing the associated public health risks worldwide is far from being reached due to multiplicity of social, political, economic, geographic, climatic, and development factors. However, a reasonable degree of health protection is attained in industrialized countries owing to their scientific, administrative, and financial capacities allowing them to use high-tech agricultural management systems. Less fortunate situations exist in equatorial and sub-equatorial developing countries mainly practicing traditional agriculture managed by smallholders for subsistence, and where the climate is suitable for mould growth and aflatoxin production. This situation worsens due to climatic change producing conditions increasingly suitable for aflatoxigenic mould growth and toxin production. Accordingly, it is difficult to harmonize the regulatory standards of aflatoxins worldwide, which prevents agri-foods of developing countries from accessing the markets of industrialized countries. To tackle the multi-faceted aflatoxin problem, actions should be taken collectively by the international community involving scientific research, technological and social development, environment protection, awareness promotion, etc. International cooperation should foster technology transfer and exchange of pertinent technical information. This review presents the main historical discoveries leading to our present knowledge on aflatoxins and the challenges that should be addressed presently and in the future at various levels to ensure higher health protection for everybody. In short, it aims to elucidate where we come from and where we should go in terms of aflatoxin research/development.

## 1. Introduction

Moulds have long been raising health issues in humans, animals as well as in plants; and they continue to be of major concern to public health and a considerable burden to the worldwide economy [1]. In addition to mycoses, they produce a myriad of poisonous toxins (mycotoxins) causing debilitating acute and chronic diseases in humans and animals. Moulds and their toxins have been the source of recurring disasters throughout the history of the mankind, but the identification of moulds as living organisms able to grow and interfere with human, animal, and plant health remained a mystery for a long time. When no causal relationship between the presence of pathogenic moulds and their adverse health effects could be established, superstitions and religious beliefs replaced rational and realistic explanations. 

The awareness of mycotoxin impact on public health and economy has drastically increased since the early 1960s with the discovery of aflatoxins following an outbreak that caused a massive poultry death in England [2]. The incident stimulated collaborative research between scientists, avian producers, and professional organizations from different countries to identify the causal agent and reveal its toxicological features in an attempt to design efficient control and preventive means. Impressive progress has been made within the first years following the poultry intoxication outbreak, leading not only to the precise identification and chemical characterization of the mycotoxins involved, i.e., aflatoxins, but also to the development of reliable analytical techniques and toxicity tests, which contributed significantly to the emergence of mycotoxicology as a standalone discipline. 

Since then, scientific research has been intensified to identify as many as possible mycotoxins with their producing fungi and the ecological conditions for their production. Studies on the toxicity patterns of mycotoxins and the extent of food and feed contamination have also been widely conducted to determine the exposure and associated health risks. While the carcinogenicity of aflatoxins to animals was established concomitantly with their discovery, a heated debate has been ongoing for more than two decades before the International Agency for Research on Cancer (IARC) recognises aflatoxin B1 (AFB1) as a human carcinogen [3].

The advent of powerful analytical tools and computer-assisted equipment at the fine point of sophistication [4] accelerated the detection and characterization of new mycotoxins leading to a fulgurant increase in the number of the naturally produced mycotoxins and toxic metabolites derived thereof. Newer types/forms of mycotoxins continue to be identified as natural fungal secondary metabolites (emerging mycotoxins) or as modified mycotoxins (conjugated and masked mycotoxins), representing further challenges for their detection, toxicological properties, and the evaluation of associated health risks on humans and animals. Despite the scientific progress in the field of mycotoxicology, in general, there is still much to do to reach the ultimate goal of developing efficient and cost-effective preventive and control means ultimately aiming at reduction of the associated health risks to the lowest possible level.

This review retraces step-by-step the historical events and discoveries that have led to the present knowledge on aflatoxins, as the most potent and widespread mycotoxins attracting the highest attention of food safety specialists, food and feed producers, stakeholders, and consumers as well as international and regional organizations. It also discusses the many challenges that remain to be overcome at the scientific and practical levels in order to reduce their present and future economic and health burden. 

## 2. Before and After Aflatoxin Discovery

### 2.1. Early Knowledge of Fungal Toxigenicity

Crop contamination with toxigenic moulds and associated health problems are as old as the settlement of mankind with the advent of the first agricultural revolution in the new stone (Neolithic) age some 12,000 years ago [5]. Plant domestication and the practice of agricultural activities raised the need for storage of harvested crops, especially grains, which were exposed to uncontrolled fungal contamination due to the lack of technical knowledge and the use of inadequate facilities. Consequently, unexplained devastating disease outbreaks struck repeatedly, causing massive deaths of humans and domestic animals without suspecting the implication of contaminated grains [6,7]. While infested grains were described in Sumerian clay tablets as early as 1900–1700 BC, the first authenticated Chinese writing reporting on the use of ergot in obstetrics dates back to 1100 BC [7]. An Assyrian tablet describing fungal contamination of grains as a “*noxious pustule in the ear of grain*” 600 years BC was probably the first reference to a cereal-borne toxic principle, although it was considered as a cereal disease with no inference to its living nature [7,8]. Because of the ignorance of the microbial world and its relation to the safety of foods and feeds, these diseases were believed to be god’s punishment for sins or demonic possessions. The notorious “witch-hunt” trials of Salem (Massachusetts, USA) in the late 17th century, where innocents were executed on an account of being the spiritual instigators of a mysterious deadly disease [9], retrospectively suspected to have been ergotism [6], is an illustration of the prevailing misinterpretations of such diseases. Ergotism caused by the ingestion of alkaloids via ergot-sclerotia contaminated rye grains or bread derived thereof is a significant milestone of such events whose causative agent has long remained a mystery. In the Middle Ages, recurrent epidemics of ergotism have been documented, mainly in continental Europe, with an accurate description of two distinct forms of the disease: convulsive and gangrenous [10]. Although the number of victims of these epidemics remains uncertain due to confusion between the gangrenous form of ergotism and bubonic plague, it is generally admitted that deaths were counted in the thousands [8,10]. While the causal link between the disease and ergoty rye grains was established in 1630, the specific responsible agent was only identified in 1853 as *Claviceps purpurea* by the French mycologist Louis-René (Edmund) Tulasne [11]. About a decade later, a preliminary characterisation of *C. purpurea* toxic principles was reported by Wenzell [12] who separated two impure fractions (ergotina and ecbolina) from spurred rye and demonstrated their alkaloid nature as well as some of the physiological effects they exert on humans. By the turn of the 20th century, a pure ergot alkaloid (ergotinin) was obtained in a crystalline state, although later reported to be devoid of pharmacodynamic activities [13]. By the first half of the 20^th^ century, a series of studies pioneered by Barger and Carr [14] purified and characterized different bioactive ergot alkaloid molecules demonstrating that they are lysergic acid derivatives [8,15,16].

A number of other mycotoxicosis episodes have been recorded throughout history in different regions of the world where they induced high mortality in human and animals, thereby dramatically affecting the economy and social life. Acute cardiac beriberi, alimentary toxic aleukia, equine leucoencephalomalasia, stachybotrotoxicosis, and Balkan endemic nephropathy are some of the remarkable outbreaks associated with fungal intoxications that have been documented. Apart from the negative social and economic impact of these diseases, they have paved the way to our current knowledge on mycotoxins, as the driving force for joint efforts to understand the diseases, determine their aetiologies, and eventually design efficient treatments or control means. As a result, some of the latter diseases have been eradicated or are well controlled to minimize their impact on public health. The research history of major mycotoxicoses and related discoveries were thoroughly reviewed previously [3,6,17], and they will not be further considered in this review, which will focus henceforth on aflatoxins.

### 2.2. Aflatoxins as a Group of Chemically Related Poisons: The Birth of Mycotoxicology

The discovery of aflatoxins in the 1960s following an outbreak of “turkey-X” disease, so-called because of its unknown nature and aetiology, was the turning point that has led to the emergence of the modern discipline of mycotoxicology. In 1960, the disease appeared in poultry farms located in a circumscribed area of London (UK), where they caused the death of more than 100,000 turkeys fed on rations containing imported Brazilian groundnut [2]. To tackle the incident, W.P. Blount, director and chief poultry advisor of the affected farms, conducted intensive field and laboratory investigations whose findings and conclusions were published the following year [2]. Although his endeavour failed to determine the causative agent involved, it provided a sound basis for peer researchers to make fairly rapid progress in reaching the goal. Notably, the author established the causal link between the toxicity of the feed and the disease; he accurately described the symptoms and lesions induced in target organs (mainly the liver); and he ruled out the involvement of infectious agents (bacteria and viruses) as well as potentially toxic chemicals commonly encountered in poultry feed as contaminants, as ingredients or additives, or as a result of deceptive practices. Meanwhile, the disease continued to cause deaths among turkeys, ducklings, and pheasants in the same area of London, specifically targeting the farms supplied from mills run by the same company that incorporated the Brazilian groundnut in feed formulations [18]. This observation urged interested parties to focus on the Brazilian nut as the main suspect. Indeed, its toxicity was further confirmed by feeding trials on turkeys, ducklings, chicken, cattle, sheep, pigs, and rats which, upon post-mortem examination, showed similar lesions, with the liver being consistently and most severely damaged [2,18,19,20,21,22,23]. However, the animals displayed different sensitivities in terms of the toxic doses, mortality rates, onset of symptoms, and severity of lesions depending on the species and the age, with ducklings being the most sensitive and rats the most resistant; and the younger the animals, the more sensitive they were. Interestingly, among poults, chickens were the most resistant, as was substantiated by feeding trials and by the observation that they were the least massively affected by the outbreaks [20]. The outcome of these studies has definitely established the toxicity of the Brazilian groundnut meal and its implication in the X disease, as was corroborated by the improvement of the situation when the Brazilian groundnut was withdrawn from feed formulations [23,24].

A significant achievement that paralleled the above findings, was the development of a reliable and fairy rapid biological technique for quantitative toxicity testing, owing to the successful preparation of a concentrated suspension of the toxin from the incriminated groundnut samples [22,25]. This afforded the availability of suitable material which could be conveniently administered *per oral* or intraperitoneally to animals at specific doses to gauge the extent of toxicity and have a response in relatively short time. The preparation procedure consists of extracting the toxic groundnut in hot methanol and suspending the dry residue derived therefrom in water for fractionation with chloroform. The dried residue of the chloroform fraction was then suspended in a methanol-water solution, defatted with petroleum ether, and distilled to remove methanol. A 250-fold concentrate of active toxin was hence recovered as an aqueous suspension after methanol evaporation, which could be orally administered to ducklings at different doses for quantitative toxicity testing [22]. Nonetheless, the toxic principle in the groundnut remained unknown at this point, and the only indication to its chemical nature was that it differs from the naturally occurring alkaloids of ragwort plant (*Senecio*) [25]. The latter toxicant was known to be associated with poultry poisoning that elicits indistinguishable symptoms and lesions from those of the “X” disease [26]. Since groundnut samples from different origins were not all toxic [23,27] and most of the toxic samples contained dead or live hyphae [18,24,28], it was assumed that the toxin is a fungal metabolite rather than an inherent poisonous constituent of groundnut [22]. 

In the meantime, X disease was also reported in Kenya as the cause of substantial losses in ducklings fed on rations containing groundnut exhibiting evident mould growth [20]. Mould contaminants were isolated and sent to the Central Veterinary Laboratory at Weybridge (England) where they were identified as *Aspergillus flavus* Link ex Fries [18]. Selected isolates were grown in laboratory media and the cultures extracted with chloroform before separation by paper chromatography developed in *n*-butanol-acetic acid. One out of eight tested extracts showed a spot with an R_f_ of 0.7 fluorescing blue under UV light. The corresponding extract was orally administered to a-day-old ducklings that it killed within 24 h, eliciting the typical symptoms and liver damage of X disease [18]. Given the low purity of the extract, it was suggested that the blue fluorescing spot contains more than one toxic metabolite [29]. This fraction was then further resolved by thin-layer chromatography (TLC) on alumina plates revealing two distinct fluorescent spots under UV light; one fluorescing blue at an R_f_ of 6.0 and the second fluorescing green at a slightly lower R_f_ [30]. It was, therefore, concluded that at least two toxic metabolites of *A. flavus* are involved in X disease and were designated “aflatoxins” referring to the producing mould species (“a” for *Aspergillus* and “fla” for *flavus*), and the letters B or G were added to differentiate between the types of toxins on the basis of their fluorescence colour under UV light (“B” for blue and “G” for green). 

The improvement of separation methods by chromatography has played a pivotal role in the progress of research on aflatoxins. These techniques provided key tools to prepare aflatoxins in a crystalline state for more precise characterization of their chemical properties and reliable toxicity testing [29,31]. Crystalline aflatoxin was obtained by cultivating toxigenic *A. flavus* under controlled conditions in synthetic laboratory media or in sterile crushed groundnut [29,30]. In both of the latter studies, recovery of crystalline aflatoxin from the cultures in synthetic media was relatively easy and straightforward. It could be achieved by direct extraction of the culture with chloroform, after which the extract is concentrated and chromatographed on silica gel column in chloroform/ethanol. The fraction fluorescing in blue under UV light was dried and the residue recrystallized from chloroform/ethanol. Conversely, the recovery of crystalline aflatoxin from cultures on crushed groundnut was rather tedious, as it required different steps of extraction, fractionation, and purification using various solvent systems and chromatography techniques. To this end, Nesbitt, O’Kelly, Sargeant [30] followed the previously described procedure leading to the 250-fold concentrate suspension [22] and continued by extracting the suspension with chloroform, concentrating the chloroform extract and separating the concentrate by column chromatography on silica gel. The fluorescent fraction was recovered from the column, dried *in vacuo* and the residue recrystallized from benzene or methanol. Aflatoxin B (AFB) and aflatoxin G (AFG) contained in the resulting crude crystalline aflatoxin were then separated from each other by 200-transfer counter-current distribution in an appropriate solvent system. The LD_50_ of AFB was determined to be 20 μg on a-day-old ducklings. Elementary, mass spectroscopy, and absorption spectra analyses assigned to AFB and AFG the respective formula C_17_H_12_O_6_ and C_17_H_12_O_7_, molecular weights 312 and 328, and maximum fluorescence peaks 429 and 450 nm. A similar approach was developed simultaneously by Van Der Zijden, Koelensmid, Boldingh [29], with some differences in solvent systems and purification steps, to yield amorphous colourless platelets designated FB1 whose toxicity was confirmed on a-day-old ducklings giving an LD_50_ of 30–50 μg [29]. Physicochemical characterization of FB1, revealed striking similarities with the AFB identified previously by Nesbitt, O’Kelly, Sargeant [30], notwithstanding some differences in the physical properties (e.g., melting temperature and infra-red, nuclear magnetic resonance, and ultraviolet absorption spectra) that were explained by impurities possibly present in each of the preparations. Indeed, a chloroform extract concentrate obtained from liquid culture of toxic *A. flavus* and fractionated by ascending TLC resolved into at least 12 different compounds [31]. Five of these compounds, three fluorescing dark-blue and two blue-green under UV light, tested hepatotoxic to ducklings. The authors noted that additional discrete fluorescent compounds may be present in the extracts, but they should be separated by as yet to be developed techniques. In an attempt to improve the technique, a concentrate of chloroform extract obtained according the latter procedure [31] was derivatized by treatment with Girard’s T reagent and its decomposed derivative fractionated by two-dimensional TLC showing a complex pattern of fluorescent spots [32]. Two of these spots fluorescing blue-violet and violet under UV light, designated FB1 and FB2 respectively, prove to be highly toxic to duckling embryos (100% mortality). FB1 was recrystallized from chloroform/ethanol yielding a pure crystalline toxin for further characterization. Results of magnetic resonance, elementary analysis, and mass spectrometry determined the MW and formula as 312 and C_17_H_12_O_6_, respectively. Comparison of the results from different studies on the characterization of aflatoxins revealed that FB1 [32], B [30], and B1 [31] isolated directly from toxic Brazilian groundnut, in addition to FB1 purified from a culture of *A. flavus* in liquid media [29] were all the same toxin, presently known as aflatoxin B1 (AFB1).

To further improve the purity of aflatoxins, a crude crystalline aflatoxin prepared from a culture of toxigenic *A. flavus* in sterile crushed groundnut was fractionated by column chromatography and purified in chloroform containing 0.25% methanol. After removal of methanol, four closely related aflatoxins were recrystallized separately from different solvents (Table 1). These aflatoxins were shown to be hepatotoxic to a-day-old ducklings to different extents, with AFB1 being the most toxic (LD_50_ of 30 μg) followed by AFB2 (LD_50_ of 60 g), while AFG1 and AFG2 were far less toxic and a dose higher than 200 μg was necessary to kill the birds after 4 days [33]. This study also established the main chemical properties of the different aflatoxins (Table 1) and demonstrated that AFB1 and AFG1 are dihydro-derivative precursors of AFB2 and AFG2, which could be prepared in vitro by chemical hydrogenation of their respective precursor molecules. Based on the above chemical characterization complemented by other chemical reactions and nuclear magnetic resonance (NMR) spectrum, Asao, Buchi, Abdel-Kader [34] and van Dorp, van Der Zijden, Beerthuis [35] reported the molecular structures of AFB and AFG as difuranocoumarin derivatives.

To study the fate of aflatoxins after ingestion, a series of trials was conducted on different animals given aflatoxin-containing rations and their excretion fluids (urine and milk), organs (e.g., liver, kidney), and product (eggs for poults) were analyzed for the presence of the aflatoxins or their derived metabolites [36,37,38,39,40]. Allcroft and Carnaghan [36] first reported that milk drawn from cows fed on toxic groundnut elicited a toxicity pattern in ducklings similar to that of the ingested toxic feed, and most of the toxin that they called “milk toxin” precipitated with the casein fraction of the milk. To further explore this observation, de Iongh, Vles and van Pelt [40] analyzed milk powder obtained from cows fed on concentrate rations containing highly toxic groundnut. Samples of the milk powder were extracted with different solvents and the final extract subjected to TLC on silica gel. The chromatography plates showed the presence of a spot with the same blue-violet fluorescence as AFB1 but with a much lower R_f_ of 0.34. The blue-violet fraction was further purified and tested for toxicity on ducklings where it induced the typical bile duct proliferation of aflatoxin lesions. Apart from its secretion in milk, the latter toxin was detected in the liver, kidney, and urine of sheep that had been administered a mixture of AFB1, AFB2, AFG1, and AFG2 [38]. The “milk toxin” was then permanently assigned the designation “aflatoxin M” (AFM) referring to milk where it was first detected. For further characterization, Holzapfel, Steyn and Purchase [39] extracted AFM from sheep urine and subjected the extract to paper chromatography where it resolved into two fractions; the first fraction (M1) had the same R_f_ and fluorescence as the parent AFM, while the second (M2) fluoresced violet and had a lower R_f_ of 0.23. The same study revealed that the newly characterized aflatoxins M1 (AFM1) and M2 (AFM2) are hydroxylated metabolites of AFB1 and AFB2, respectively. 

Additional aflatoxin types have since been discovered either as naturally occurring fungal metabolites or as derivatives generated from the metabolism of parent aflatoxins that accumulate in organs and/or are secreted in body fluids. This was the case of the aflatoxin hemiketals AFB2_a_ and AFG2_a_, the respective hydroxy derivatives of AFB1 and AFG1, which were first shown not to be toxic to a-day-old ducklings after administration of 1.2 mg of each toxin [41]. In contrast, a subsequent study confirmed the toxicity of AFB2_a_ to ducklings and bacteria, although to a significantly lower extent compared with AFB1 [42]. Aflatoxin B1 could also be metabolized by monkey and excreted in the urine in a demethylated form (aflatoxin P1, AFP1), now used as a biomarker to assess the exposure of humans to AFB1 [43]. At present, more than 18 types of aflatoxins with different chemical properties and toxicity patterns are known, among which at least 13 types are naturally occurring in foods and feeds that had supported the growth of aflatoxigenic moulds [44,45].

### 2.3. Causality between Aflatoxins and Liver Cancer 

While scientific research focused on the detection and characterization of aflatoxins during the early 1960s, a survey on the incidence of various types of cancer in Africa indicated that the rate of hepatocellular carcinoma (HCC) was particularly high in specific regions of the continent [46]. The author of the survey speculated that fungal toxins including the then newly discovered aflatoxins, are possible causative agents. This speculation was supported by the earlier observation that peanut was incidentally commonly consumed in the regions where liver cancer was frequently diagnosed [47,48,49]. On the other hand, peanut was known to be usually contaminated with aflatoxins at levels frequently exceeding 2 mg/kg and, in some instances, reaching 20 mg/kg in the high risk regions for liver cancer [50]. The suspicion of the association between peanut consumption and liver cancer was also supported by health issues that arose from the implementation of the United Nations Food and Agricultural Organization (FAO) initiative to control kwashiorkor in African countries. In this initiative, a-year-old malnourished children were fed meals supplemented with peanut as a protein source for 10 months at the daily rate of 70 g for an initial period of 4 months and 140 g for the next 6 months [51,52]. Two of these children were clinically evaluated 4 years after termination of the initiative and found to carry chronic liver damage [51]. Preserved remnants of peanut supplement were retrospectively analyzed for aflatoxins and found to contain between 0.5 and 1.0 mg of aflatoxin/kg meal, thereby exposing the children to a daily intake of 35 to 140 μg aflatoxin [49]. As a consequence, the FAO established a provisional limit of 30 μg/kg of peanut supplements on the basis of risk/benefit considerations as a preventive measure from chronic liver intoxication [53]. The first acute toxicity of aflatoxins causing liver failure was reported in 1967 in Uganda following the death of a teenager who had been regularly eating mouldy cassava contaminated with 1700 μg/kg of aflatoxins [54]. The post-mortem histopathological examination showed that the child’s liver tissues had undergone identical changes to those described in a monkey treated experimentally with aflatoxins [55]. 

By the end of 1960s, numerous epidemiological studies and animal trials have reported on the relationship between the exposure to aflatoxins and different types of liver cancer. Relevant reports were critically reviewed in the first volume of the IARC which recognized the widespread aflatoxin-contamination of marketed and cooked foods in developing countries, mainly in the sub-Saharan Africa and Southeastern Asia regions. The IARC then considered that the pertaining studies provide a “circumstantial evidence” for the association of aflatoxin dietary intake with liver cancer in humans [56]. Nonetheless, the direct causal relationship was explicitly excluded, essentially because of two confounding factors: (i) the concomitant presence of other mycotoxins in aflatoxin-contaminated foods, and (ii) hepatitis B (HB) virus for being endemic in the areas with high aflatoxin dietary intake and high incidence of liver cancer [56]. Moreover, by comparing aflatoxin dietary exposure estimates with the incidence of HB virus infections in different USA regions at high-risk of liver cancer, the U.S. Food and Drug Administration (USFDA) strongly supported the view that HB virus was the actual cause of the cancer and the occurrence of aflatoxins in foods was only coincidental [57,58]. However, the method used to evaluate the risk was later criticized for the lack of accuracy in the exposure estimates [59]. Nonetheless, the U.S. issued the first regulation on aflatoxins, as food contaminants, in 1965 with a maximum limit (ML) of 30 *pp*b (1*pp*b = 1 μg/L or kg product) for total aflatoxins (sum of AFB1, AFB2, AFG1, and AFG2) in foods; this ML was decreased to 20 *pp*b in 1969 and maintained until today [60]. In 1977, after the characterization of AFM1 as a hazard to consumers, the USFDA established the action level of this mycotoxin in milk to 0.5 *pp*b. 

For few decades, the causality between aflatoxins and liver cancer remained a major issue of debate internationally, while research work kept generating supportive data to reach a consensus regarding the carcinogenicity of aflatoxins. By the mid–1970s, the carcinogenicity and mutagenicity of aflatoxins have been extensively demonstrated in different animal species and in bacteria. In addition, many epidemiological studies have inferred a positive correlation between aflatoxin dietary intake and liver cancer in humans. All relevant reports available by 1975, were critically examined by the IARC working group who advised to maintain the previous status considering the outcome of the review as a “circumstantial evidence” for the carcinogenicity of aflatoxins in humans [61]. Although laboratory tests had shown beyond doubt that the administration of aflatoxins via different routes to animals, including birds, ruminants, fish, rodents, and non-human primates caused liver cancer, the IARC working group had many reservations on the rationality of epidemiological and clinical studies. This precluded the group from concluding as to the existence of a direct causal link between aflatoxins and carcinogenicity in humans. One of the major reservations was the fact that most of the cohort and case-control studies had been conducted in developing countries where the highest rate of liver cancer was recorded, which shed doubts on the accuracy of the registration, diagnosis, and completeness of the medical files. In addition, the most significant epidemiological studies have been carried out retrospectively with no certainly that the records and analytical tests were comprehensive, properly conducted, and the records well archived. Moreover, technical flaws were noted regarding the correlation studies, most of which have also omitted to take into account the incidence of chronic hepatitis B and C infections to rule out or confirm the possible interference of these major risk factors with aflatoxins in causing liver cancer [62].

The debate continued until 1987 before the IARC finally classified the “naturally occurring mixtures of aflatoxins” in the group 1 carcinogens considering the availability of new tangible data generated from better designed studies [62]. Yet, the specific role of each of HB virus infection and aflatoxins to trigger liver cancer remained to be clarified. By 1992, high-quality studies meeting the IARC provisions to circumvent the limitations raised in previous reviews, have provided “sufficient evidence” for the carcinogenicity of AFB1. Notably, a cohort study has established an almost linear relationship between the dietary intake of AFB1 and the mortality rate from liver cancer, thereby substantiating the direct implication of this particular aflatoxin in the disease [63]. It also unraveled the intricate overlap between the high incidence of HB virus infections and the high dietary exposure to aflatoxins in areas at high-risk of liver cancer by confirming their synergistic effect. The study demonstrated that the risk of liver cancer for individuals with a high aflatoxin intake increased by approximately 13-fold in seropositive individuals for HB-surface antigens (HBsAg) compared with HBsAg-seronegative individuals. Conversely, the mortality rate from liver cancer of HBsAg-seropositive individuals was 10 times lower in areas with low aflatoxin dietary exposure. In addition to the well-designed statistical analysis, the mains strengths of this study were the prospective follow-up of the cohort participants and the use for the first time of biomarkers to assess aflatoxin exposure. The biomarkers used were the AFB1 metabolites AFP1 and AFM1, and the DNA-adduct AFB1-N^7^-Gua, whose concentrations were determined periodically in the urine of the cohort members. These results were confirmed by other robust prospective cohort and case-control studies using similar approach and different biomarkers [64,65]. 

In view of the availability of new convincing studies reporting on a direct link between AFB1 dietary exposure and liver cancer, this aflatoxin was included in group 1 carcinogens in the 1992 edition of the IARC monographs [66]. However, AFM1 was classified in group 2B of “possibly carcinogenic substances” because of “inadequate evidence” of carcinogenicity in humans; whereas, it was concluded to the “lack of evidence” for carcinogenicity in humans of AFG1, AFG2, and AFB2, and to “inadequate evidence” and “limited evidence” for the carcinogenicity in experimental animals of AFG2 and AFB2 aflatoxins, respectively [66]. These conclusions were reaffirmed by the IARC in 2002, with the provision of additional confirmatory data from a number of well-designed prospective cohort studies supported by laboratory analyses using specific biomarkers of exposure [67]. In 2004, the most deleterious aflatoxicosis ever recorded worldwide (317 cases with 125 deaths) occurred in Kenya due to the consumption of highly contaminated maize [68,69]. In addition to the provision of an additional evidence of aflatoxin implication in acute intoxications, this incident revealed a high positive correlation between the levels of aflatoxin-lysine adduct in the serum and AFB1 intake [70]. Since then, this adduct has been largely used as one of the most reliable biomarkers to quantitate chronic exposure to aflatoxins. 

Mechanistic studies demonstrating that the carcinogenicity of aflatoxins arises from their genotoxic action were also taken into account as sensitive state-of-the art tools that help classify chemical agents in the appropriate carcinogenicity group. The first of such studies was conducted by Bressac, Kew, Wands [71] who demonstrated that the carcinogenicity of AFB1 relates primarily to its genotoxicity. According to these authors, AFB1 targets the tumor-suppressor gene *TP53*, also known as *p53*, where it induces a point mutation by substituting the third base G of the codon 249 for the base T (AGG to AGT), with a consequent substitution of the amino acid serine for arginine in the gene product (p53-R249S). This mutation arises from the formation of a DNA adduct, 8,9-dihydro-8-(N^7^-guanosinyl)-9-hydroxy (AFB1–N^7^-guo), via an active intermediate metabolite, aflatoxin B-exo-8-9-epoxide (AFBO), generated in the liver from AFB1 by action of a cytochrome P450 enzyme (CYP450) [64,72,73]. As this appeared to be a general mechanism among aflatoxins, AFB1, AFB2, AFG1, AFG2 and AFM1 were all classified by the IARC in group 1 carcinogens in 2012 [74]. Nonetheless, despite the well-established synergistic action between aflatoxins and HB virus to increase the risk of liver cancer, as provided by cohort studies and biomarker analysis, this synergy awaits to be mechanistically clarified [65,75,76,77,78]. The use of biomarkers, seems to be a promising means to settle definitely the long-standing debate around the specific role of each of the two major risk-factors, aflatoxins and HB virus infection, in HCC induction [79,80]. Hepatitis virus C (HC) and the blue-green algal hepatotoxic peptides, mycrocystins, which were evoked as additional risk factors that would act in synergy with aflatoxins to cause liver cancer should also be given due attention [62,81]. 

Beyond the substantiation of exposure to aflatoxins, and their mode of action at the molecular level, mechanistic studies are expected to help progressing the ongoing efforts aiming to reduce the incidence of aflatoxins in foods and feeds in order to detect liver cancer at early stages while the prevention or prognostic improvement are still possible [80]. However, selection for the most appropriate and stable biomarkers for specific purposes, and the design of easy and sensitive methods for their detection in secretion fluids, the blood and/or specific organs, remain a challenging issue for future studies [84]. This trend announces the emergence of a new era in mycotoxin research based on advanced molecular biology and nanotechnology techniques [85]. The main historical milestones discussed above that have led to our present knowledge on aflatoxins, their publication dates, and research circumstances are summarized in Table 2.

## 3. Challenges and Prospects for Aflatoxin Research

The recent advances in scientific research on toxicology coupled to the increased accuracy and sensitivity of the analytical tools helped improve our understanding on aflatoxins. However, the same advances raised new challenges that the international community should overcome to reach the ultimate goal of reducing as much as possible the incidence of aflatoxins in foods and feeds, and the consequent health risks they pose to humans and animals. Difficulties to meet this goal internationally are greater with the huge economic and technological gaps between industrialized and developing countries, which also represent major barriers to trading in the scope of the open borders advocated by the world trade organization (WTO). These challenges can be technical, ecological, socio-economic, or legislative in nature.

### 3.1. Technical Challenges

As is the case for any hazard of concern to food safety, a minimum of scientific information on aflatoxins is necessary for risk assessment studies aiming to help stakeholders adequately manage the risk. It also helps national, regional, or international regulatory authorities issue science-based legislative provisions to reduce mis/biased interpretation disputes. Such information includes, but not limited to, knowledge of chemical and toxicological properties, biosynthesis and biodegradation pathways, the producing moulds and their ecological niches, and a thorough estimation of the concentrations and the distribution in foods and feeds in a country, a region, and worldwide. The availability of reliable, sensitive, and specific analytical methods is a central element for the generation the necessary data in all the above-mentioned disciplines. Although aflatoxins are the mycotoxins that have received the most attention in this regard and tremendous amount of data has built up since their discovery, important gaps remain to be filled through further efforts on technical issues. 

#### 3.1.1. Analytical Methods and Challenges for Future Development 

There are many accurate, specific, and sensitive chromatography- and immunology-based techniques routinely used in scientific research on aflatoxins or in regulatory laboratories for official control purposes. Novel methods, such as those using biosensors and optical-based systems intended for rapid in-field and laboratory use to quantitate, semi-quantitate, or screen for aflatoxins are also being increasingly used awaiting improvements in their accuracy and sensitivity to be fully validated [4,86]. The conventional chromatography- and immunology-based methods are the most reliable and widely used but they are costly, time-consuming, or require skilled personnel to be operated [4]. Therefore, they are not readily accessible to developing countries where the problems of aflatoxins are the most severe [87,88]. Due to the global concern of aflatoxins, which can possibly cross borders via international trade, it is urgent to develop easy-to-use, low-cost, and yet reliable methods to meet the needs of smallholder farmers, and small-and-medium enterprises (SME) of food and feed processors in developing countries and elsewhere. They can afford to monitor the levels of aflatoxin-contamination of their produce and take corrective actions to reduce the contamination when necessary. At the same time, this will generate data for meaningful determinations of exposure as a key element for accurate risk assessment, an approach necessary to issue science-based food safety standards. These techniques can also be used in official laboratories to perform efficient and cost-effective controls for proper enforcement of national regulations. Rapid methods of emerging analytical technologies using kits or portable devices such as biosensors and optical-based systems appear to be the best candidates to fulfill such a goal, but they require further refinements to be validated for quantitative analysis [4,89,90,91]. Although the accessibility to analytical methods cannot guarantee, by itself, efficient reduction of aflatoxin contamination that requires laborious efforts at different levels, it is a prerequisite to any action to be successfully undertaken. It also provides an important yardstick for producers to monitor aflatoxin levels and appraise the efficacy of the quality assurance approach they may adopt to mitigate the incidence of aflatoxins. Only such dynamic can permit gradual reduction in aflatoxin contamination in the most affected countries to support periodic updates of the national regulations in the perspective of global harmonization of food safety standards advocated by the sanitary and phytosanitary (SPS) agreement [92,93].

Beyond the level of development of a country or a region, recent discoveries of emerging mycotoxins or those that had been chemically modified during processing or as a result of microbial, animal, or plant metabolism (conjugated or masked mycotoxins) raise new challenges in terms of detection and quantification [94,95]. Moreover, the number of mycotoxin-derived metabolites and mycotoxin precursors that continues to grow with the advance of metabolomic studies adds to these challenges [91]. Apart from the putative or confirmed toxicity of these compounds, they may act in synergy to increase toxicity of the classical mycotoxins, including aflatoxins. Co-occurrence of emerging and modified/masked mycotoxins with regulated mycotoxins is a common phenomenon in agricultural commodities; and a typical commodity contains seven to 75 different mycotoxins, with an average of 30 [94,96,97]. Failure to detect all mycotoxins and their metabolites in food and feed may result in misdiagnosing mycotoxicoses or underestimating the associated health risks [97]. 

From the about 18 known types of aflatoxins, only AFB1, AFB2, AFG1, AFG2, and AFM1 are presently regulated in some commodities in a number of countries; and they are analyzed by conventional techniques. The other aflatoxins are overlooked from the regulatory standpoint despite their potential toxicity or ability to convert into their toxic parents. For example, aflatoxicol that contaminates milk and dairy products has the same toxicity as aflatoxin M1 and is readily converted into its highly toxic parent aflatoxin B1 in the liver [98,99]. Although parasiticol is weakly mutagenic and probably non-carcinogenic, it has the same acute toxicity as B1 and may be of concern to food safety [100]. Similarly, many aflatoxin precursors, such as those of AFB1, were shown to be toxic to different extents [101]. Yet, these toxins are not subject to routine analysis in foods and feeds, nor are they detected by the conventional methods. Furthermore, despite the lack of data on the interactions (antagonism or synergy) of the emerging mycotoxins with aflatoxins, their detection and quantification remain crucial to have a clear picture of such interactions and for accuracy of risk assessment studies. 

In view of the above considerations, intensive work has been done during the last decade to develop methods capable of detecting multiple analytes of different nature (e.g., mycotoxins and their metabolites, pesticides, fungicides, and veterinary drug residues, and plant toxins) in one sample analysis. Many state-of-the-art technologies are being considered to develop multi-mycotoxin analysis with simplified sample preparation and a validated “fit-for-purpose” status [95]. Development of such multi-analyte/multiplex techniques based on liquid chromatography (LC) coupled with mass-spectrometry (MS) or with high resolution mass-spectrometry (HRMS) received increased attention to address the challenge. The first validated of such techniques was LC coupled with a tandem MS (LC-MS/MS), which could simultaneously analyze quantitatively 39 different mycotoxins, including emerging and masked mycotoxins, as well as derived-metabolites in maize and wheat [102]. Based on this technology, Biomin Holding GmbH (Erber group, Getzersdorf, Austria; https://www.nationalhogfarmer.com) has developed multi-mycotoxin analytical devices, e.g., the Spectrum 380®, which can analyze simultaneously more than 450 different fungal metabolites, encompassing all of the known mycotoxins and their derived metabolites [91]. A fit-for-purpose, cost-effective LC-MS/MS multi-mycotoxin method, validated for 13 different mycotoxins, including aflatoxin B1, was recently applied to survey the multi-mycotoxin occurrence in maize and wheat produced in South Africa [103]. Computer-assisted techniques using LC-HRSM was suggested to be the most promising LC-MS-based technology; it has the same performances as the LC-MS/MS with an additional advantage of allowing retrospective analysis to screen for mycotoxins that had not been regulated at the time of sample analysis [60]. In addition, this technique can be fit to metabolomic studies for the detection of thousands of low molecular-weight metabolites in a wide range of concentrations in a single analysis [91]. The emerging “omic” discipline of metabolomics is, therefore, expected to reveal novel secondary metabolites of moulds not yet known, thereby extending the repertoire of mycotoxins to include non-targeted novel mould metabolites that can be either toxic or detoxified derivatives. This is achieved by the stable isotype labeling (SIL) technique whereby a biological system, e.g., a plant, is treated with a native mycotoxin and its uniformly labeled ^13^C counterpart (1:1). The fate of the precursor is then traced by LC-HRSM to generate data and process them by an appropriate software [104]. Applied to wheat artificially inoculated with deoxynivalenol (DON), this technique revealed the presence of eight novel DON derivatives, suggested to have resulted from a detoxification strategy of the plant [105]. Although no such studies, to our knowledge, have been done on aflatoxins they are strongly encouraged to provide a comprehensive view on the constitutive members of this important group of natural toxicants and its biological detoxification. Indeed, metabolomics can also be fit for gene-function studies to relate, for example, the genetic inheritance of the ability of an organism to metabolize/detoxify a mycotoxin. This can in turn be useful for the development of decontamination strategies of foods and feeds. 

Despite the high performances of the above discussed techniques and the undeniable potential they have, as avant-garde technologies, to advance analytical science, their high cost (equipment and reagents) and their requirement for highly trained personnel limit their affordability by developing countries as is currently the case of conventional techniques. In addition, they are facing technical limitations mostly related to the management of matrix effects, such as: 

The need to perform tedious multiple clean-up steps prior to the analysis for detection, identification, and quantification, depending on the intended use of the analysis, 

The need for calibration to ensure that the concentrations of all mycotoxins in a sample fall within their respective ranges (i.e., linearity of the calibration curve for all mycotoxins to be determined). A procedure that can be laborious given the wide variations in concentration ranges of the mycotoxins in a sample (ng/mL for some mycotoxins vs. μg/mL for others). The extract should then be concentrated or diluted of as appropriate, usually leading to compromises at the expense of the sensitivity. Uniformly labeled mycotoxins for internal standards have been successfully used to circumvent this limitation, but this was faced the availability and affordability. Limited availability and high cost of reference standards for external and internal calibration are especially crucial impediments for detection and quantification of emerging and modified mycotoxins,

Cross-talk across assays resulting from the interference between signals of different mycotoxins in the same sample may hinder mycotoxins with weak signals, leading to falls negative results. 

These limitations and the strategies to cope with them have been thoroughly reviewed previously [60,91,95,106,107], and there is a general agreement on the absence of a universal solution to eliminate the matrix-effect in all cases. Therefore, this aspect represents one of the major challenges that researchers will be facing in the coming years to increase the sensitivity, accuracy, repeatability, and efficacy of the LC-MS-based techniques for multi-mycotoxin analyses with a minimal sample preparation. It was suggested that overcoming these limitations may revolutionize the techniques to make them fully automated with a minimum human errors [60]. Multi-mycotoxin analysis using multiplex assay kits such as those described for drug development may also be a feasible and affordable solution, but they also need improvements for the accuracy, sensitivity, and specificity to be validated for intended purposes [108]. 

#### 3.1.2. Dosimetry of Aflatoxins and Risk Prediction with Biomarkers

The science-based provisions of the SPS agreement enforced in 1995 emphasize the central role that risk assessment should play in food safety regulations (Article 2.2 of the SPS agreement) to promote the global trade while maintaining an appropriate level of protection (ALOP) of health and life [93,109]. Consequently, research on the dietary intake of hazards has been accelerated for quantitative determination of health risks as a basis to determine the tolerable daily intake (TDI) and thereby set the maximum limit of contaminants in foods and feeds. Aflatoxins were among the first chemical hazards whose health risk was assessed and the outcome used to issue or revise regulatory standards in different countries as well as in the *codex alimentarius* (CA)*,* the benchmark for international trade. However, the accuracy and completeness of the published data remain hampered by the high uncertainties and inconsistencies in the estimations of food consumption and the levels of food contamination with aflatoxins; the two crucial parameters for dietary exposure estimation [88]. This holds especially true for developing countries where the diet consists mainly of self-produced and traditionally made foods of doubtful sanitary quality and that are usually neither declared nor controlled for sanitary quality before consumption [88]. Besides, most of the good quality foods produced in these countries are exported while those that fail to meet the safety standards and those that are not controlled are marketed locally via unformal routes, thereby increasing uncertainties with a tendency to underestimate the exposure. An alternative epidemiological approach using biomarkers in biological fluids, mainly blood and urine, has been developed and is gaining increased interest [88]. The main aim of this approach is to develop and validate biomarkers for quantitative estimation of exposure to aflatoxins and associated health risks. Biomarkers can also be useful to predict the risk of disease/cancer development ahead of the onset to allow the implementation of preventive measures such as chemoprotective strategies or diet change [110]. Table 3 summarizes the main biomarkers presently known with their limitations and strengths. 

**Figure 1:**
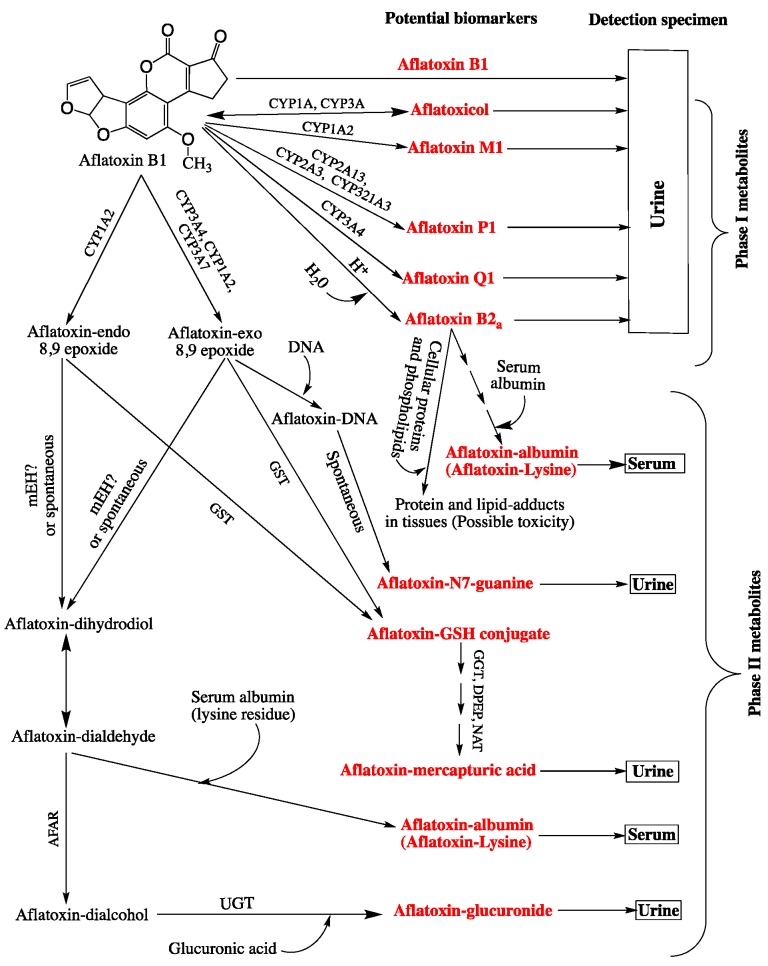
Fate of aflatoxin B1 in the liver showing the metabolites with potential to be used as biomarkers “in red boldface letters” for exposure determination and the biofluids where they can be detected and quantified. For the details on the formation of aflatoxin-albumin adduct from aflatoxin B2a, see Figure 2 below. Adapted from [146]. *Abbreviations*: CYP: Cytochrome P450 enzymes; GGT: γ-glutamyltranspeptidase; DPEP: Dipeptidase; NAT: Nacetyltransferase; UGT: UPD-glucuronosyltransferases, mEH: Microsomal epoxide hydrolase; GST: Glutathione-S transferase; AFAR: Aflatoxin aldehyde reductase; ?: No sound evidence to whether or not mEH is necessary for the transformation of aflatoxin-exo 8,9 epoxide into aflatoxin-dihydrodiol.

**Figure 2:**
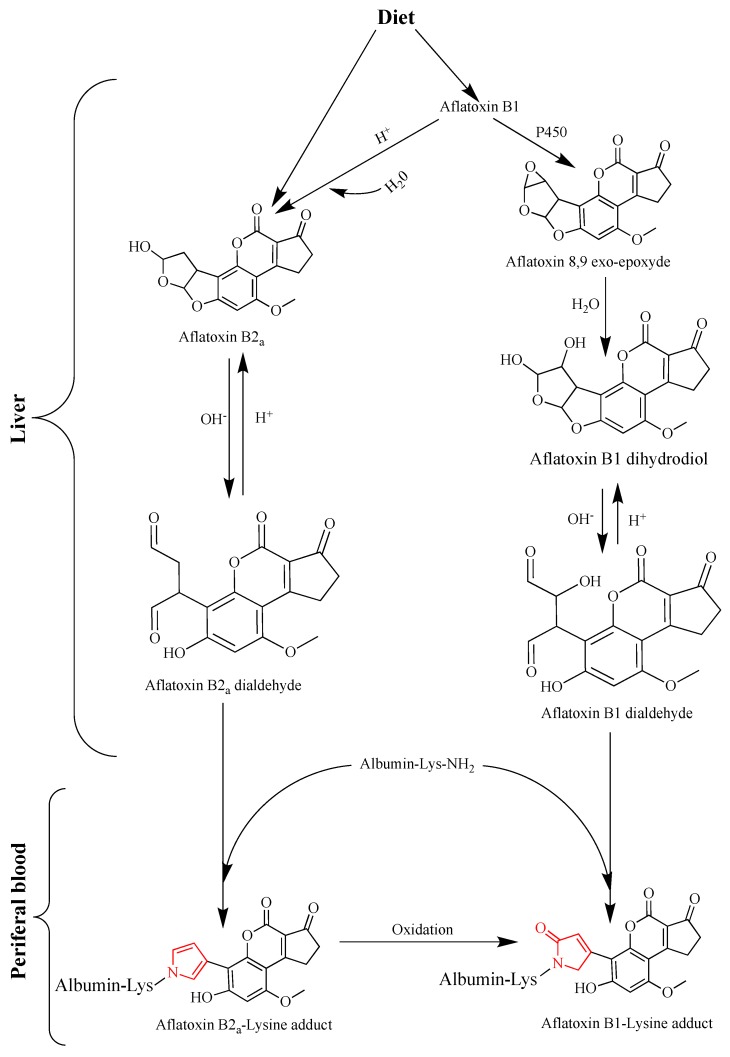
Comparative formation pathways of aflatoxin B1-Lys (lysine) adduct deriving exclusively from aflatoxin B1 (Right) or from aflatoxin B1 and B2_a_ (Left) as precursors. The pyrrole and pyrrolin-2-one rings that characterize aflatoxin B2a- and aflatoxin B1-albumin adducts are drawn in red. Adapted from [116].

Currently, aflatoxin-albumin adduct in blood serum is the most reliable biomarker for a long-term exposure to AFB1 owing to its longest half-life (20 days on average) compared with any other known urinary adduct, and it persists in the serum for more than 3 months [84,111]. A highly positive correlation between aflatoxin dietary intake and the level of aflatoxin-albumin adduct in human serum has been repeatedly demonstrated in humans and animals; and a percentage of 1.4 to 2.3 of ingested aflatoxin was shown to bind covalently serum albumin [112,113]. A series of studies using this adduct as a biomarker demonstrated a strong dose-response relationship between aflatoxin exposure in utero or during early infancy and growth impairment [114]. Nonetheless, the significance of this biomarker as a molecular dosimeter for quantitative risk assessment or as a predictive parameter to identify individuals prone to develop HCC was questioned [115]. The controversy arises from the fact that the metabolism of AFB1 via the genotoxic epoxide formation pathway is not the only source for the generation of aflatoxin-albumin adduct [116]. In the genotoxic pathway (Figure 1), this adduct derives from the metabolism of AFB1 in the liver where it is first oxidized by cytochrome P450 enzymes (CYP3A4, CYP1A2, and CYP3A7) to form AFB1-exo-8,9-epoxide (the highly reactive genotoxic metabolite) and AFB1-endo-8,9-epoxide (less active). These unstable epoxides are spontaneously transformed into AFB1-8,9-dihydrodiol which undergoes a base-catalyzed ring opening to aflatoxin-dialdehyde that in turn reacts by Schiff base formation with the lysine side chain of serum albumin to form the AFB1-lysine adduct (Figure 1 and Figure 2). This pathway leads to the formation of the adduct that can adequately inform on AFB1 exposure and its potential to induce HCC. However, aflatoxin-albumin adduct was recently suggested to also derive via an alternative pathway from AFB2_a_ [116]. Accordingly, AFB2_a_, from the diet or as an AFB1 metabolite, is directly oxidized to AFB2_a_-dialdehyde and reacts by a dual condensation with serum albumin to form AFB2_a_-albumin adduct with a pyrrole ring, contrary to aflatoxin B1-albumin adduct that has a typical pyrrolin-2-one ring [116]. Under oxidative conditions, the pyrrole ring of the aflatoxin B2_a_-albumin adduct is oxidized to yield the adduct (with a pyrrolin-2-one ring) as that formed via the genotoxic pathway directly from aflatoxin B1 (Figure 2). In the light of these findings, the authors concluded that aflatoxin-albumin adduct may not always be a reliable indicator of aflatoxin B1 intake and its subsequent metabolism through the genotoxic pathway. This is consistent with an earlier report on the lack of significance of serum albumin-adduct levels for quantitative risk determination and for accurate prediction of the risk for HCC development [115]. The lack of correlation may be further enhanced by the fact that the aflatoxin-dialdehyde does not react entirely with serum albumin and part of it is reduced with aflatoxin aldehyde reductase (AFAR) into aflatoxin-dialcohol, which then reacts with glucuronic acid under the action of a UPD-glucuronosyltransferase to be detoxified in the form of aflatoxin-glucuronide and excreted in the urine (Figure 1). Moreover, part of the AFB2_a_ produced in the liver binds covalently to cellular proteins and phospholipids (Figure 1) instead of binding albumin [116]. These various pathways and fates of precursor and intermediate metabolites make the quantitative correlation between the ingested AFB1 and the resulting aflatoxin-albumin adduct dependent on different factors that drive the metabolic reactions in favor of one or another pathway. Nonetheless, aflatoxin-albumin adduct is currently the most reliable biomarker which was validated to determine aflatoxin “chronic” exposure. 

Aflatoxin-N^7^-guanine is an aflatoxin-DNA adduct that has also been intensively used as a biomarker to estimate recent exposure to aflatoxin and, hence, to diagnose acute aflatoxicosis, or identify individuals or groups of people at high risk of liver cancer [117]. This adduct results from the metabolism of AFB1 in the liver involving different cytochrome P450 enzymes (e.g., CYP3A4, CYP1A2, CYP3A7) to form the highly reactive intermediate metabolite AFBO, which binds covalently to the N^7^ of the DNA guanine residue forming the 8,9-dihydroxy-8-(N^7^) guanyl-9-hydroxy (AFB1-gua). The AFB1-gua is unstable due to electric charge interactions within the DNA molecule and is, therefore, rapidly released from the DNA leaving an apurinic site. The free adduct is then excreted exclusively in the urine where it serves as an exposure and risk biomarker [118]. Alternatively, it is stabilized on the DNA by opening the ribose ring to form an AFB1-formamidopyrimidine (AFB1-FAPY), which can thus be useful to measure the effective biological dose and, hence, for dose-response characterization and disease outcome. The concentrations of both of the latter aflatoxin-DNA adducts (AFB1-gua and AFB1-FAPY) in tissue samples taken from the liver and kidney of mice were shown to be proportional to the administered levels of AFB1 [119]. A highly positive correlation was also demonstrated in humans between AFB1-gua adduct in the urine and the dietary intake of AFB1 [65,110,117]. However, the major limitation of the latter metabolite as a biomarker resides int that it can only inform on a recent intake of the aflatoxin or be used to monitor exposure changes in individuals subject to interventions, such as chemoprevention treatment or exposure-avoidance through a specific diet [115,117,120]. Despite the evident advantages AFB1-FAPY as a risk biomarker, the accessibility to tissue samples is a major limitation and has, so far, been done only in experimental animals or post-mortem histopathological examinations. Both AFB1-gua and AFB1-FAPY were suggested to be used for histopathological diagnosis to relate the etiology of liver cancer to aflatoxins [121]. 

Aflatoxins B1 and its metabolites excreted in the urine are also routinely used as biomarkers (Figure 1). The so-called phase I metabolites of AFB1, such as AFM1, AFP1, AFQ1, aflatoxicol, and AFB2_a_ produced in the liver by various cytochrome P450 enzymes (Figure 1) have been considered as exposure biomarkers for internal dose determinations and for possible correlation between their levels and risk of HCC development. The excretion of AFM1 and AFP1 was first demonstrated in the urine from mice fed on AFB1-contaminated rations [122]. The study also reported these metabolites (AFM1 and AFP1) were excreted in urine from humans exposed to AFB1 through the diet, and a dose-response relationship between AFM1 and HCC has been established in cohort studies in Taiwan [123]. Also, the amount of AFM1 in the urine of individuals from the aflatoxin-endemic Guangxi region of China was correlated with AFB1 intake, and a proportion of 1.23 to 2.18% of dietary AFB1 was excreted in the urine as AFM1 [124]. The presence of AFP1 in urine samples was directly related to the risk of liver cancer [63], but no linear relationship between its levels in the urine and exposure to AFB1 could be demonstrated [110]. Meanwhile AFQ1 was rarely detected in urine samples from people or animals fed on AFB1-contaminated foods or feeds [122]. 

Phase II metabolites, other than DNA and protein adducts discussed above, namely aflatoxin-glucuronide and aflatoxin mercapturic acid derived from aflatoxin-dialcohol and aflatoxin-GSH conjugate, respectively (Figure 1) are additional potential urinary biomarkers that have received a limited attention [80]. However, the usefulness of aflatoxin mercapturic acid as an indicator of the effectiveness of chemoprevention strategies has been emphasized [80,84,120,125]. Similarly, aflatoxin-glucuronide would serve the same purpose, but more importantly, it could provide a practical protective means against AFB1 toxicity by the administration of AFAR inducer drugs. This highly inducible enzyme (AFAR) catalyzes aflatoxin-dialdehyde reduction into aflatoxin-dialcohol, which does not form adducts proteins/albumin but is rather detoxified into the non-toxic aflatoxin-glucuronide conjugate [126]. This prevents the formation of aflatoxin-albumin adduct from aflatoxin-dialdehyde and favors the detoxification of AFB1 over the genotoxic pathway. Induction of AFAR by natural or synthetic antioxidants and other drugs was demonstrated in rats and shown to increase their resistance to AFB1 [80,127,128]. 

Other AFB1 metabolites have a potential for use as risk biomarkers and for the biologically effective dose measurements to provide reliable indication on the exposure and a dose-response effect. These are, for example, AFB1-exo-8,9-epoxide and AFB1-FAPY, directly involved in the mechanism of toxicity but since they are not excreted in biological fluids, their levels should be determined in tissues of target organs, which is not always feasible. There is, therefore, a need to develop specific and safe sampling and analytical procedures to take advantage of these intermediate metabolites that would provide valuable and precise information to establish the dose-response effect (i.e., genotoxic dose) necessary for quantitative risk assessment. Other molecular biomarkers in this category may be identified in the future, as metabolic pathways and pharmacokinetics of aflatoxins are being progressively elucidated. 

According to Groopman and Kensler [110], the validation of a biomarker should undergo a stringent procedure for its suitability to provide pertinent information spanning from exposure to the outcome of a disease and be experimentally tested for sensitivity, specificity, accuracy, and reliability through pilot studies on humans, among other provisions. Under these conditions, few biomarkers meet individually all the required validation criteria. As matter of fact, the challenge for efficient use of biomarkers to assess and predict the risk of cancer development in individuals, communities, and larger-number populations has been raised for over two decades [110] and little progress has been made since then due to limitations of each of the presently known biomarkers (Table 3). The same authors suggested an alternative approach consisting of using composite sets of biomarkers, each of which addresses specific criteria so that whole group of sets meets the overall validation requirements. Therefore, it seems more appropriate for future studies to investigate the combinations of biomarkers that would provide complementary information to have the most accurate and precise indication on the extent of exposure to predict the disease risk and outcome [129,130]. Success of this approach is contingent to the availability of appropriate and easy-to-use analytical techniques that can apply to all biomarkers in a composite set at once. Presently, immunology- and/or chromatography-based techniques are the most used to quantitate biomarkers individually and they should usually be adapted to a specific biomarker under specific conditions to fit-for-purpose. ELISA, radioimmunoassay (RIA), HPLC with fluorescence (HPLC-Fl) detection, LC-MS and LC-MS/MS, LC-HRMS, and atomic mass spectrometry (AMS) are frequently used, each of which bears advantages and limitations [114]. Again, liquid chromatography coupled with isotope dilution tandem mass spectrometry and LC-HRMS were recently shown to be the most accurate, precise, specific, and sensitive [131,132]; yet, the cost and the need for skilled personnel to perform the analyses are their main drawbacks. To this end, the development of multianalyte techniques, discussed above, specifically designed to quantitate as many biomarkers as possible in one analysis, taking into account the matrix effect, warrants consideration. Therefore, the next challenge that research on aflatoxins will be facing, in this particular issue, is to find the most appropriate set of biomarkers to be used together and the most reliable techniques for their detection and quantification. 

### 3.2. Natural and Socioeconomic Challenges

Aflatoxin contamination of foods and feeds is unevenly distributed throughout the world depending on the geographical zones and the prevailing climatic conditions. Countries in the tropical and sub-tropical zones with hot and humid climates located between 40° North and 40° South of the equator are the most exposed to the growth of aflatoxigenic fungi and aflatoxin production in agricultural products [147]. Developing countries in this region, especially those of Southeast Asia and South Africa, have been known for the highest incidence of aflatoxins and rates of related HCC worldwide [1,148]. Sustained efforts involving international cooperation have been made to alleviate the health and economic burden associated with aflatoxins in these countries [149]. However, the efforts have been hampered, in part, by the favorable climates in these countries to the growth of aflatoxigenic molds and aflatoxin production, which become ideal with occasional drought followed by flooding episodes [69,70]. Under these conditions, aflatoxin contamination of agricultural products can only be controlled by modern high-tech agricultural practices using integrated approach from pre-harvest to consumption. Such an approach is not affordable by farmers of the region, most of whom are smallholders practicing subsistence agriculture with traditional management systems [150,151]. A common practice among smallholder farmers consists of dividing the harvest into three main parts; one for domestic consumption (including animal feed), another saved as seed for the next year’s sowing, and the third sold to intermediary dealers who generally store it inadequately for 2 months or more before retailing to make the highest profit. Poor storage conditions and the absence of official inspection and control by the government authorities provides greater opportunity for aflatoxin accumulation. Abnormally high levels of aflatoxins have been reached in less than two months of storage under faulty conditions and have occasionally caused major aflatoxicosis outbreaks [152,153,154,155,156,157]. The lack of awareness about the impact of aflatoxins on crop yield and safety from the producers and consumers alike is another hurdle that has been attributed to the poor risk communication and the absence of functional bridges between academic and development institutions [158]. Fostering risk communication on adverse health effects of aflatoxins in foods and feeds is incumbent to the governments of these countries as part of political commitment to promote food safety on the basis of risk analysis (RA) approach. This approach that has been strongly recommended by international organizations (e.g., FAO, WHO, and WTO) as the scientific basis for food safety regulatory provisions, considers risk communication as a key component aimed to increase public awareness and stipulates that it should involve all interested parties, including consumers, industry, non-governmental organizations (NGOs), academia, media, etc., with the government participation and supervision [159]. 

Sophisticated weather monitoring systems and the development of reliable predictive models are increasingly necessary to forecast unusual climate events and seasonal variations ahead of their occurrence to take preventive measures when possible [160]. The situation is expected to worsen in the future with the ongoing climatic change and its impact on the extent and emergence of new and/or modified food safety problems, including mycotoxicosis, worldwide. Using predictive modeling to measure aflatoxin risk index (AFI/ARI), defined as the potential of *A. flavus* to interact with crop and produce aflatoxin, the mean AFI associated with AFB1-contaminated maize in Europe was estimated to increase during the next 100 years with global warming scenarios of 2 °C and 5 °C by 92% (AFI = 73.25) and 140% (AFI = 95.09), respectively compared with the present status (AFI = 38.20) [160]. According to the study, maize cultivation and its contamination with aflatoxins will expend throughout Europe to reach currently spared high altitudes zones between 45° and 60° North of the equator, while Southern Europe countries are expected to be at high risk with levels of aflatoxins exceeding the legal limits more frequently than is currently the case. A retrospective simulation of peanut contamination with aflatoxin in Australia revealed a three-fold increase in the average ARI recorded during the period of 1980–2007 compared with the previous period from 1980 backward to 1891; the increase in the ARI was associated with the increase in ambient temperature and decrease in rainfalls [161]. For practical applications by farmers, different ARI-based models have also been developed to predict crop contamination with aflatoxins in response to temperature and water stress within a given season [160,162,163,164]. Although ARIs were determined under different ecological factors (soil parameters, insects infestations, crop cultivar, etc.) and by using different approaches and software, they all converge to the conclusion that aflatoxin contamination tends to increase with increased temperature (within the range of aflatoxigenic molds growth; 11.5–42.5 °C) and decreased rainfalls; the typical indication of climate warming. There is a consensus that ARI-based models will increasingly be used to map high risk areas globally and monitor in-season risk for aflatoxin contamination to allow producers determine the harvesting time that minimizes contamination. This appears to be plausible, as the ARIs were demonstrated to correlate well with the variations of aflatoxin contamination of different crops [161,163]. However, most of these models have received a limited application, as they have not been validated in the field, or they were fit for specific agro-climatic areas but not for others [165,166,167,168,169,170,171]. In contrast, “agricultural production system simulator” (APSIM) developed in Australia was validated for peanut aflatoxin contamination in the field under tropical and sub-tropical climates ([161] and is gaining popularity worldwide [162,163,164,172]. APSIM is a modelling framework consisting of different modules that simulate biophysical processes in farming systems to generate economic and ecological outcomes to help manage climatic risks [172]. It includes peanut and maize modules which inform on aflatoxin contamination in response to drought and temperature from sowing to pre-harvest [161,163,173]. APSIM module for peanut was put into practice to assist growers determine the harvest time via an interactive web-based decision support (www.apsim.info/afloman) [161].

The climate change impact on aflatoxin contamination of crops is of more concern in tropical and sub-tropical countries, already facing serious problems of aflatoxin contaminations, with the El Niño-Southern Oscillation (ENSO) phenomenon associated with interannual extreme shifts in rainfall and temperature causing alternating drought and excess rainy periods. As a result, ecological conditions are modified to favor the growth of microbial pathogens with a consequent increase and emergence of foodborne diseases [174]. The increase in food and feed contamination with aflatoxins and the occurrence of aflatoxicosis outbreaks subsequent to severe drought followed by unseasonal heavy rains is well documented [70,147,175,176,177]. Climatic change was suggested to affect the expression of the regulatory genes *AflR* and *AflS*, as well as the early structural gene *AflD* in *A. flavus* and *A. parasiticus*. The expression of these genes is significantly influenced by ecological stress factors, mainly temperature and water availability; highest production of AFB1 was demonstrated to occur at a temperature/a_w_ combinations of 25 °C/0.95, 25 °C/0.99, 30 °C/0.95, and 30 °C/0.99 [178]. With the ongoing climatic change, such environmental conditions for aflatoxin production are likely to be common in many regions throughout the world. During the dry period, the temperature exceeds 25 °C, and the excess rain that usually follows increases the water activity to above 0.95 providing ideal conditions for aflatoxin production. Unless robust measures with modern high-tech and management systems are taken, crop contamination with aflatoxins will undeniably tend to increase. A concern that can be addressed by the adoption of integrated management systems (IMS), such as the 2018 version of the International Organization for Standardization (ISO) ISO 22000 which encompasses environment management system (ISO 1401) in addition to three other major management systems: quality assurance (ISO 9001), food safety (ISO 22000, version 2005), and occupational health and safety (ISO 45001). This strongly suggests, that in additions to control means for aflatoxin contamination at the production, in-farm storage, and market levels, there is a need to curb the climatic change as the driving factor. Therefore, one of the most challenging issues that scientific research/development on aflatoxins will be facing for many years to come is probably the impact of climatic change on aflatoxin contamination of foods and feeds. It is a multifaceted issue that requires immediate and anticipatory actions with high degree of coordination between education/research and development institutions, international cooperation, various organs of the United Nations, NGOs, etc. Appropriate monitoring of climatic change using sophisticated analytical and modeling tools with the provisions of regulatory measures to prevent environment pollution or allow its rehabilitation is necessary and involves joint international efforts. Moreover, research and development programs should use innovative ways of partnership between scientists, developers, and funding institutions for the highest possible impact of the results in the field. In particular, the involvement of social scientists as key players in any research project on aflatoxins have been suggested as an imperious need to bridge scientific findings and the reality of the social context [158]. Governments may as well adopt specific policies to encourage farmers and food- and feed-producing industries improve the safety of produce sensitive to aflatoxin contamination, such as grains and derivatives, by the adoption of a preferential pricing based on aflatoxin content. Nevertheless, this measure should be considered with caution when applied in developing countries under the present social, political, and economic conditions. Firstly, because it implies commitment from the government, food and feed industries, and/or traders to perform aflatoxin determinations on a routine basis and on a large number of samples of different products, which may incur unaffordable extra-costs and require unavailable analytical facilities (see Section 3.1.1); and secondly, by doing so, many samples may be found unfit for human consumption and should, therefore, be redirected to animal feeding or discarded from the food chain with consequent food security issues. Additionally, such a measure may encourage corruptive practices by operators who would manipulate the analytical results. In fact, this measure that has been successfully used for many years in industrialized countries for raw milk pricing could not be applied in developing countries despite its theoretical practicability, and low cost and ease of the required analyses. Difficulties in its implementation lay essentially with socioeconomic reasons and government apprehension of shortage in milk supply due to the likely extensive non-conformities to regulatory standards. The situation is certainly more complicated for aflatoxin contamination of foods and feeds, and may rather require technical and financial incentives (tax deduction on agricultural inputs, equipment, and machinery, creation of cooperatives, availability of resistant cultivars, etc.) from the government to encourage the transition toward the practice of modern agriculture, including drying and storage.

### 3.3. Actions to Mitigate Aflatoxin-Induced Health Risks

The ultimate goal of scientific research on aflatoxins is the development of efficient means to alleviate the health risk they pose to humans and animals. Despite the tremendous efforts that have been done to reduce the incidence of these natural toxicants in foods and feeds since their discovery, they are still widely distributed at high levels in nature and continue to raise serious public health concerns. They are also one of the main barriers to international trade of agricultural products, especially between developing and industrialized countries. Actions to improve the situation can be undertaken at two main levels: (i) at the food and feed level by reducing their contamination and hence the dietary exposure, and (ii) at the consumer level by adopting strategies to prevent or retard the onset of the diseases, especially the HCC, in populations or individuals at high risk [80].

#### 3.3.1. At Commodities’ Level: Reducing the Dietary Intake

It is well established that safe decontamination of foods and feeds from aflatoxins is a real challenge, and that only preventive integrated holistic approaches involving quality assurance systems can yield satisfactory results in reducing the risk associated with aflatoxins. Actions should be undertaken at all the production stages; from pre-harvest to post-harvest, including irrigation systems, selection of resistant cultivars (natural selection or genetically modified crops), pest control, monitoring climatic conditions, managing the time of harvest, drying techniques, transportation, conditioning, and storage (in-site, in the market, and at home) [179,180]. While this approach can reasonably be followed in industrialized countries, it is not affordable for developing ones where agriculture and livestock production are mainly practiced for subsistence by low-income smallholder farmers. [181] Similar situation applies to the food and feed industries predominated by SMEs and SMIs with insufficient capacity to afford high technology requirements. To avoid losses caused by disposal of agricultural products deemed to be unfit for consumption for their high aflatoxin content, numerous detoxification/decontamination techniques using biological, chemical, and physical treatments have been designed to ensure “safe” levels of contamination before consumption [180,182,183,184,185,186]. Depending on the product, some of these treatments prove efficient in removing or detoxifying aflatoxins from/in certain foods and feeds [184]. Yet, to find practical applications, a treatment should possess other features, including low cost, easy to use, no partial toxicity retention or generation of new toxic compounds, and no adverse effects on the nutritional or sensory quality of the product. These characteristics are rarely met in a single treatment, which can, in addition, apply to all commodities and for all known aflatoxins. On the other hand, the impact of these strategies at the global level remains far below expectations due to the wide technological and economic gaps between developing and industrialized countries. Beside the ongoing research/development programs on detoxification/decontamination means of foods and feeds, additional efforts should be made to foster international cooperation in order to allow developing countries implement quality assurance programs and good agricultural practices. Nevertheless, this essential prerequisite to make a real progress in this issue globally can only be achieved on the long run and depends largely on the implementation of adequate politics to promote international development. 

#### 3.3.2. At the Consumer Level: Chemoprotection/Chemoprevention

Under certain conditions, aflatoxin contamination cannot be significantly restricted in foods and feeds most of which remain highly contaminated while their condemnation would seriously compromise food security. This is true for most developing countries where some extent of unsafe levels of aflatoxin contamination must be accepted providing intervention strategies are applied to interfere with the toxicity or limit the bioavailability of aflatoxins. 

##### Reducing the Risk by Interfering with the Toxicity of Aflatoxins

**Figure 3:**
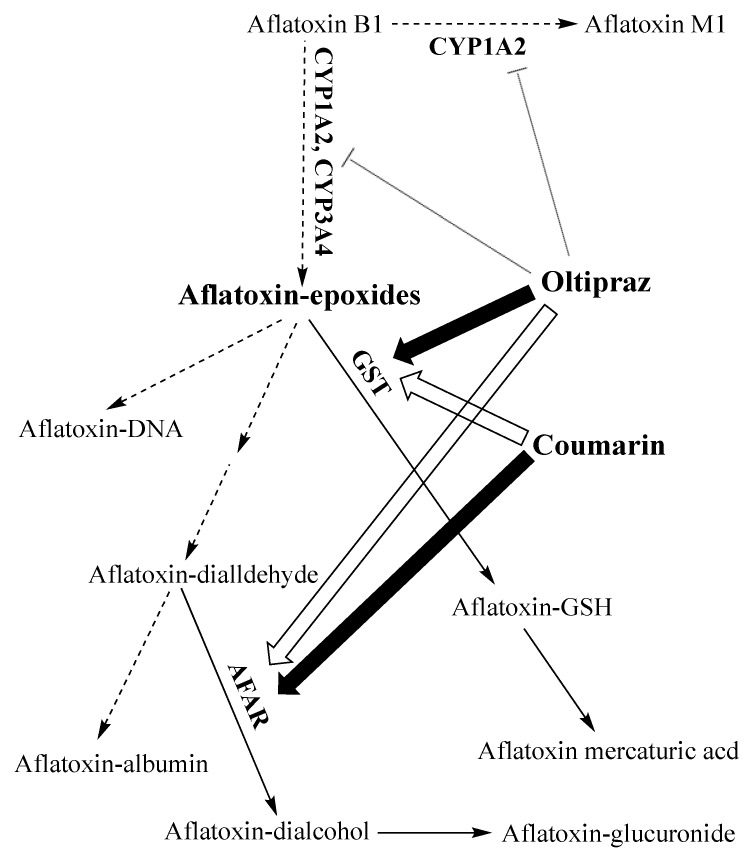
Action of oltipraz and coumarin on the metabolism of aflatoxin B1 against aflatoxin-induced hepatocarcinogenicity. The dominant action of each agent is indicated by a large filled arrow while a lesser inducing effect is indicated by large hallow arrows; T-shaped lines indicate inhibition of the enzymes; dashed arrows indicate reduced rate of the reaction; plain arrows indicate the routes favored by the action of the inducers [127,143]. (For abbreviations, see captions of Figure 1).

To express their toxicity in humans or animals, aflatoxins should first be activated to form reactive aflatoxin-exo- and -endo-epoxides by the action of mono-oxygenase cytochrome enzyme systems (CYP P450). These products, especially the highly reactive aflatoxin-8,9-exo-epoxide, bind nucleic acids (RNA, DNA) forming adducts, predominantly aflatoxin-gua, that are directly involved in mutagenicity and carcinogenicity. Otherwise, the epoxides undergo the pathway forming adducts with proteins and phospholipids via the intermediate metabolite, aflatoxin-dialdehyde, to exert toxicity by interfering with the functionality of these macromolecules (Figure 1). Conversely, the epoxides are detoxified, mainly via glutathione-S-transferases (GTS) or AFAR mediated conjugation with glutathione or glucuronic acid, respectively. Based on these mechanisms, chemoprotective strategies have been developed with the use of phase II enzyme-inducers to shift the equilibrium of the reactions towards detoxification endpoints (e.g., aflatoxin-GSH, aflatoxin-mercapturic acid, and aflatoxin-glucuronide) that will ultimately be excreted in urine, bile, and/or feces. Various natural and synthetic compounds, including phenolic antioxidants, indoles, isothiocyanates, coumarins, flavones, allyl sulfides, dithiocarbamates, dithiolethiones, and triterpenoids analogues were shown to modulate the metabolism of carcinogens by this mechanism [143]. Many of these compounds exert a chemoprotective effect against AFB1-induced liver tumors through the induction of the genetic expression and catalytic activities of phase II enzymes [127,187,188] (Figure 3). Oltipraz (4-methyl-5-(2-pyrazinyl)-1,2-dithiole-3-thione), a synthetic derivative of the natural dithiolethione, is one of these compounds that has received the greatest interest. Its chemoprotective effect has been extensively demonstrated in animals and was shown to be mainly related to the induction of GTS activity as evidenced by the decrease in aflatoxin-albumin and aflatoxin-gua adducts in the serum and urine, respectively [115,189,190,191,192,193]. This chemoprotective agent also inhibits phase I cytochrome P450 enzymes, mainly CYP1A2 and CYP3A4 [143,194], resulting in a decrease of aflatoxin-epoxides and AFM1 levels (Figure 3). The positive outcome of animal studies has encouraged clinical trials with oltipraz in the Qidong region of China where exposure to aflatoxins and HCC incidence are among the highest in the world. Oral administration of oltipraz to people at high risk in this region demonstrated its ability to drive the metabolism of AFB1 towards the detoxification pathway, providing a proof of principle for the aflatoxin-detoxifying effect of this drug in humans [144]. The administration of oltipraz at a daily rate of 125 mg induced a 6-fold increase in the excretion of aflatoxin-mercapturic acid in the urine compared with placebo controls, indicating a significant induction of GST. No such a trend in aflatoxin-mercapturic acid excretion was observed when the drug was administered at 500 mg weekly, suggesting that high doses of oltipraz inhibit phase I enzyme, thereby restricting epoxide formation and hence the subsequent steps of the pathway (Figure 3). 

Another synthetic drug of high potential for chemoprotection intervention against aflatoxin-induced HCC is the triterpenoid analogue of oleanolic acid, oleanane triterpenoid 1-[2-cyano-3-,12-dioxooleana-1,9(11)-dien-28-oyl]imidazole (CDDO-Im). This drug reduced the formation of preneoplastic lesions in F344 rats (inbred strain commonly used in carcinogenicity lifetime bioassays for its high susceptibility to carcinogens) challenged with AFB1 (25 μg/rat/day) by 85% and more than 99% at doses of 1 and 100 μmol/kg body weight (bw), respectively, showing 100-fold higher potency than oltipraz [195]. The drug also induced 40% to 90% reduction of DNA adduct levels in treated rats (AFB1 + CDDO-Im) compared with control rats [AFB1 + vehicle (10% dimethyl sulfoxide, 10% Cremophor-EL, and PBS)] in a dosage-dependent manner. Additionally, the study reported that CDDO-Im increased significantly the concentrations of mRNA transcripts of genes involved in phase II metabolism (e.g., *GSTA2*, *GSTA5*, *AFAR*, and *EPHX1*) within 6 h of administration by gavage. Microarray analysis of phase II and antioxidant gene expression in knock out and wild-type strains, demonstrated that CDDO-Im induces the genes through *Nrf2*-mediated signaling pathways, suggesting that, in addition to its anti-tumorigenic property, the drug may also act as a potent antioxidant, anti-inflammatory, apoptotic, and cytoprotective agent [195]. A more recent study conducted on the same rat strain (F344) by using a risk-reduction cohort approach, confirmed the remarkable potency of CDDO-Im as a chemoprotective agent and its superior efficacy compared to oltipraz [196]. In this study, rats challenged with a daily dose of AFB1 (200 μg/kg bw) for 4 weeks, were given 30 μmol/kg bw of CDDO-Im (treated rats) three times a week starting one week before the first AFB1 dosage and continuing throughout the remaining 4 weeks of treatment; control rats were given vehicle instead of the drug as in the previous study [195]. The effects of the CDDO-Im were measured by analyses of AFB1 biomarkers in the urine (GST conjugate and AFB1-gua), GST-P positive foci (presumptive preneoplastic phenotype) and AFB1-DNA adducts in the liver, and RNA expression signature genes that characterize AFB1 DNA damages in rats [196]. Results revealed that CDDO-Im provided complete protection (0 case on 20 test rats) against CCH, whereas 96% (22/23) incidence was observed in control rats; the hepatic burden of GST-P positive foci was absent in the treated rats, but increased from 0 to ~14% in controls over the four weeks of AFB1 dosage. Regarding biomarkers, a three-fold increase in the concentration of AFB1-N-acetylcysteine, a detoxification derivative of aflatoxin-glutathione conjugate, was observed after the first dose of AFB1 and maintained thereafter; AFB1-gua adduct concentration in the urine of control rats was seven-fold higher than in the urine of treated rats after the fourth week of treatment, with an overall reduction of 66%. CDDO-Im abrogated almost totally the toxicogenomic RNA expression of 7 discrete signature genes providing a molecular evidence for the inhibition of AFB1 genotoxicity and CCH initiation. Moreover, DNA analysis by isotope dilution mass spectroscopy revealed that, despite a significant reduction of AFB1-DNA adducts in the liver tissue of treated rats, a steady-state burden of AFB1-DNA adducts, predominantly FAPy, remained in the liver of treated rats but without exerting any harmful effects [196]. FAPy is known to be the most mutagenic AFB1-DNA adduct [197]. The latter observation coupled to the complete ablation of CCH and the absence of signature RNA transcripts, led the authors to conclude that AFB1 has a threshold genotoxicity, i.e., a given number of lesions per DNA molecule below which AFB1 causes no adverse health effects. This clearly argues against the prevailing “non-threshold” view stipulating that there is “no safe” level for AFB1; 1 molecule can cause some harm (linearity) [191,198,199]. The study has attracted wide interest from peer scientists with both admiration and criticism not only for its rational experimental design and for providing an additional proof-of-principal for the high potency of CDDO-Im and potential to ensure complete protection against CCH, but also for reviving the debate on the “no-threshold/linear” vs. “threshold/non-linear” dose-response paradigms in quantitative risk assessment of genotoxic hazards. Nonetheless, the claim about the non-linear dose-response relationship at very low doses and the applicability of the results to humans have raised some reservations [200,201]. While recognizing the valuable contribution of the study to the advance of knowledge in the field, Eaton and Schaupp [200] scrutinize the study and expressed skepticism about some interpretations. In particular, they consider that further evidence is needed to soundly establish the non-linear dose-response and wonder why the authors did not determine the threshold level from their own results. They argue that the complex mechanism of the Nrf2/Keap1/ARE signaling pathways that mediate CDDO-Im anti-tumorigenicity may also activate other cellular processes, e.g., anti-inflammation, and eliminate or interfere with the progression and promotion of initiated cells resulting in an “apparent” threshold, which may not be valid for chronic exposure to very low doses of AFB1 in the absence of CDDO-Im. On the contrary, Olden and Vulimiri [201] supported the arguments given by Johnson, Egner, Baxter [196] for the non-linearity of the dose-response relationship. Moreover, Eaton and Schaupp [200] questioned the putative chemoprotective efficacy of CDDO-Im in humans as was demonstrated in rats. As a general mechanism of action in both rats and humans, CDDO-Im activates the *Nrf2*/*Keap1*/*ARE* pathways which, in turn, upregulate the expression GST genes whose products (glutathione-S-transferases) catalyze the conjugation of glutathione to the AFBO to produce non-toxic conjugates. However, rat GST genes involved in *Nrf2*-mediated aflatoxin detoxification code for GST isoforms, e.g., class α GstA5, that are far more active in detoxifying the toxic aflatoxin epoxide than the human isoforms, e.g., class mu GTSM [200]. This controversy can only be settled by clinical trials involving people at risk from endemic zones, such as the Qidong and Guangxi regions of China, as has been done for oltipraz [144]. It should be emphasized, however, that before treating humans at high risk with CDDO-Im, it is worthwhile to perform a preliminary screening, using appropriate biomarkers, for possible individuals with undiagnosed CCH to whom the prescription of this drug would be a contraindication. The activation of Nrf2-dependent signaling pathways prevents the initiation and progression of tumor in normal or pre-cancerous tissues but, on the contrary, increases chemoresistance of fully malignant cells and promotes tumor growth [202].

Dietary supplementation of selenium was also suggested as a chemoprotection intervention due to its demonstrated effectiveness to protect various animal species against AFB1-induced hepatotoxicity, immunotoxicity, and genotoxicity [203,204,205,206,207]. However, the mechanism of action of this micronutrient remained unexplained despite some studies suggesting its role in enhancing the antioxidant capacity of cells [206,208] and interfering with CYP450 enzymes responsible for AFB1 activation [209]. A recent study demonstrated that the supplementation of poultry feed with selenium prevented efficiently AFB1-mediated liver injury and dysfunctions [210]. The authors demonstrated that selenium has a dual action in the prevention of liver injury: (i) Inhibition of CYP450 isozymes involved in AFB1 transformation into to the active epoxide isomer (AFBO), and (ii) Increase of antioxidant capacities of cells through the upregulation of selenoprotein gene coding for antioxidant proteins [211], thereby reducing the oxidative stress responsible for various adverse health effects including mutagenicity, immunotoxicity, and carcinogenicity. This chemopreventive intervention could also be envisaged for humans, but it needs to be considered carefully regarding the amount of selenium to be supplemented, as relatively high doses of this micronutrient cause severe toxicities [212].

Despite the proven efficacy of drug-based chemoprotection strategies using natural or synthetic inducers of phase II-enzymes in mitigating the toxicity of aflatoxin, its practical implementation to large-number populations face significant challenges. The use of drugs as part of the regular diet represent a burden to the household budget, especially in target resource-poor settings in developing countries. Additionally, such a practice is perceived by consumers, who lack awareness of the high risk they incur, as a culturally unacceptable deviation from their culinary habits. To address these challenges, government actions aiming to ensure compliance and deliberate involvement of populations are required through the allocation of budgets for incentives and subsidies, organization of training sessions, education, and effective communication campaigns to increase awareness [213]. This is essentially a political decision that may not be feasible or sustainable in many developing countries for insufficiency of budgets and/or the ranking among national priority action plans. 

Gradual introduction in the diet of specific foods or food-extracts rich in natural inducers of aflatoxin-detoxifying enzymes may be feasible and culturally more acceptable in most countries, especially with the new trend of consumer preferences for drug-free, diversified, and natural foods. Foods rich in bioactive phytochemicals (e.g., glucosinolates, sulforaphanes, polyphenols, and ascorbic acid) are promising sources to substitute drug-based chemoprevention strategies [149]. Green tea and members of the cruciferous vegetable family are good candidates owing to their high contents in these compounds [214,215]. The detoxifying action of phytochemicals on AFB1 through the induction of phase-II detoxifying enzymes and the inhibition of the aflatoxin-activating phase-I enzymes have been demonstrated in animals as well as by clinical trials [216,217,218]. 

Green tea polyphenols (GTP) were demonstrated to have an anti-aflatoxin mutagenic effect on *Salmonella typhimurium* [214] and to inhibit the initiation of aflatoxin-induced hepatocarcinogenesis in rats [219]. Surveys have reported an inverse relationship between the level of green-tea consumption and the risk of cancer development in humans [220,221]. A clinical trial involving highly exposed residents to aflatoxins from Guangxi Zhuang Autonomous region of China who were given commercial GTP at two doses (500 or 1000 mg) or placebo four time daily for three months showed that the treatment resulted in a significant increase of aflatoxin-mercapturic acid concentration in urine after the first month, suggesting a decrease in aflatoxin-DNA adduct formation [222]. Further clinical studies are, nonetheless, needed to be conducted on a larger scale to confirm the extent of chemoprotective action in humans, safety, and the form and dose of delivery with regard to the well-known instability of polyphenols and their anti-nutritive properties due to interactions with proteins and micronutrients [223,224]. 

In broccoli, sulforaphane (4-methylsulfinylbutyl isothiocyanate), a well-recognized potent anticarcinogen, was also demonstrated to deviate the metabolism of aflatoxins towards the detoxification pathways by upregulating GTS enzymes [217]. Broccoli is particularly rich in glucoraphanin, the precursor of sulforaphane (SFN) that it releases upon the action of myrosinases [225,226]. The high potency of SFN in preventing cancer diseases [216,217,218] makes it a favorite candidate for plant-based chemoprotection strategies against aflatoxin-induced hepato-carcinogenicity. Among cruciferous vegetables, broccoli is by far the main source of this phytochemical compound whose precursor glucoraphanin can be extracted by simple water infusion owing to its hydrosolubility [227]. This precursor (glucoraphanin) can be transformed into the active SFN by the action of myrosinases, also present in the plant. Three main ways have been suggested for the application of SFN in chemoprotection strategies:

A broccoli-rich diet to increase the dietary intake of glucoraphanin which is converted into active SFN in the gastrointestinal (GI) tract by the action of the accompanying myrosinases or by gut microbiota. However, for a maximum benefit, broccoli should be eaten raw. Cooking destroys the heat labile myrosinases and the activation of glucoraphanin will then depend solely on the gut microbiota. This raises bioavailability issue due to the inconsistent activation of the glucoraphanin depending on individuals’ microbiota that varies greatly with the age, health status, medications possibly taken in conjunction with the intervention, diet, etc. [228]. In addition, the content of glucoraphanin in broccoli also varies highly with the cultivar and variety, the stage of maturity, the part of the plant eaten (stem or sprout), and storage conditions [215]. This leads to inconsistent and unpredictable results of SFN-based chemoprotection strategy. 

Use of water infusions from young sprouts (3-days old) as the easiest and cheapest extraction way while offering a practical means of delivery as a drink for a given number of times during the day. Here again, the instability of myrosinases to heat during infusion in hot water is a limiting factor leading to complete reliance on the gut microbiota for the activation and bioavailability, and hence to inconsistent outcome.

Use of food supplement preparations containing pure lyophilized SFN obtained by enzymatic treatment of broccoli infusions with myrosinases [227,228]. Due to the high reactivity and instability of SFN, stabilization treatments are required to preserve its biological activity and bioavailability upon storage under normal environmental conditions (ambient temperature and humidity). A recent study showed that SFN was stabilized by complexation with the food-grade α-cyclodextrin, and it preserved its total biological activity for extended periods at relatively high storage temperatures (22 and 37 °C) [227]. Commercial food supplements enriched with pure SFN claiming to have anti-cancer properties are already in the market, but their efficacies and means of delivery (oral, topical, or a combination of both) for optimal action are as yet to be soundly validated by clinical studies [229]. Ongoing clinical trials on SFN and those that have already been conducted with outcomes can be visited at https://www.clinicaltrials.gov (accessed 28 July 2019). 

Other phytochemicals may have potential applications in plant-based chemoprotection strategies against aflatoxin-induced liver cancer, but they have been poorly investigated or are still to be discovered given the increased scientific interest in curative or prophylactic phytotherapy. For example, coumarin, a natural benzopyrone, was shown to be a potent inducer of AFAR, GTS, and NAD(P)H quinone oxidoreductase, known for their anti-oxidative stress action [127]. The study showed that AFAR activity was increased by up to 400-fold in male rats fed on coumarin-containing rations (0.5%) surpassing the effect of two other natural chemoprotective agents, benzyl isothiocyanate and indole-3-carbinol, which caused 5- to 7-fold increase in the activity of the enzyme. Meanwhile, the same coumarin-added diet had a lesser effect, although significant, on the activity GTS that they increased by 10 to 65 times. These results suggest that coumarins may have an additive effect with GTS-inducer phytochemicals against the tumorigenic activity of aflatoxins and are hence potential candidates for multi-agent use in chemoprotection strategies. However, the interactions between different chemoprotective agents, their toxicities and side effects, and means and doses of delivery in humans and animals should to be given full attention before considering their practical application. 

**Figure 4:**
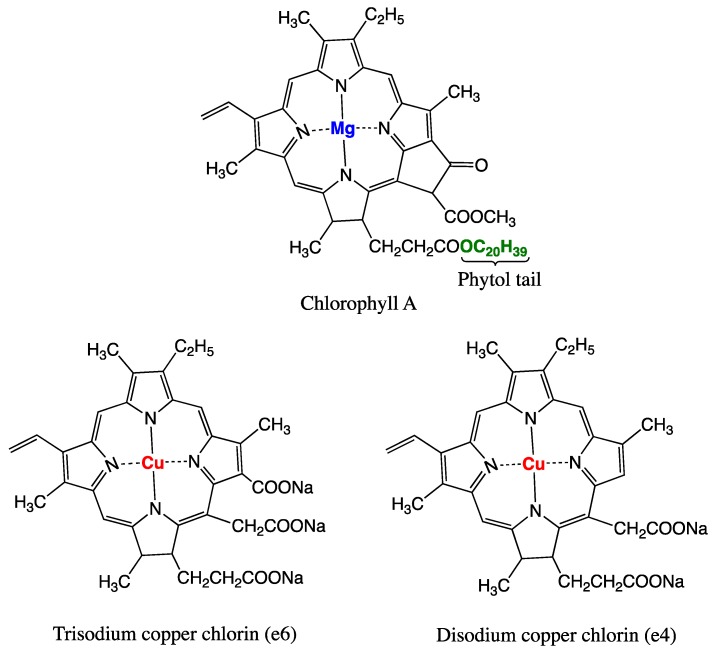
Chlorophyll A molecule naturally found in green plants, algae, and photosynthetic bacteria, and the two main semi-synthetic derivatives, disodium copper chlorin e4 and trisodium copper chlorin e6, commonly used in commercial chlorophyllin food supplements.

Another plant-based chemoprotective strategy against aflatoxin-induced cancer using chlorophyll (chl) and derivatives, such as the natural metabolites chlorophyllides (chlide) and pheophorbides (pho) [230,231,232], and the semi-synthetic chlorophyllin (chln) is attracting increased commercial and scientific interest [230,233]. The antimutagenic and antigenotoxic activities of chl have long been known, and its anti-carcinogenicity has been extensively demonstrated in experimental animals or in vitro on cancer cell-lines [231,233,234,235,236,237,238]. Chlorophyll is a ubiquitous constituent of green leafy plants and algae that form substantial components of the human diet, and a diet rich in green vegetables was suggested to provide the necessary amount of chl for an effective chemoprotection [233,237]. However, as a food supplement, the effect of chl is drastically restricted by its high instability and water-insolubility [239]. To circumvent these limitations, semi-synthetic preparations called chlorophyllins (chln) have been produced and are commercially available as food supplements under different trade names. Chln preparations are also permitted as food coloring additives under the E number of E141 (http://www.food-info.net/uk/e/e141.htm, accessed July 31, 2019). These are sodium-copper salt derivatives of chlorophyll consisting mainly of mixtures of disodium copper chlorin e4 and trisodium copper chlorin e6, which derive from chlorophyll A by substituting the magnesium ion of the porphyrin ring (chlorin) for a copper ion and removing the hydrophobic phytol tail (Figure 4). 

This provides chlorophyllin with the desired stability and water solubility while keeping the functional properties of the parent chlorophyll. *In vivo* studies on chemoprotective effect of chlorophyll and chlorophyllin showed that they were both potent anticarcinogens with high potential for practical application to alleviate the liver-cancer incidence [235,236]. Chlorophyllin was shown to induce in vitro cell cycle arrest and apoptosis [238], and to exert a cytotoxic effect on various cancer cell lines, although to a lesser extent than chl and chlide [231]. The administration of 100 mg of chln three times a day for 4 months to Qidong residents highly exposed to unavoidable dietary aflatoxins, has resulted in a-55% decrease in the median urinary concentrations of aflatoxin-gua adduct compared with those recorded in placebo controls [240]. As regards the mode of action of chlorophyll and derivatives, most studies tend to concur that they act by directly complexing and trapping the carcinogen regardless of its species thereby reducing its bioavailability and systemic absorption [230,235,236,237]. Other studies, however, showed that they may act in different manners depending on the chl derivative. For example, while chlide reduced the formation of aflatoxin B1-gua adduct by trapping the toxin via the complex aflatoxin-chlide formation in murine hepatoma cells, pho additionally induced phase II GTS enzymes [230]. At the molecular level, studies on cancer cell-lines suggest that chl and derivatives act by inactivating signal transduction pathways [231,238]. 

##### Reducing the Risk by Interfering with the Bioavailability of Aflatoxins

Interference with the bioavailability of aflatoxins in the GI tract, by non-digestible food-grade sorbents or microorganisms has also been considered for chemoprotective strategy development. Enterosorption of aflatoxins by montmorillonite clay (NovaSil^TM^; NS), an anti-caking agent in animal feeds, was shown to remove efficiently aflatoxins and reduce their toxicity in rats without noticeable side effects [241]. The safety of uniform particle size of NS (UPNS) and its effectiveness in removing aflatoxins in humans were also demonstrated by clinical trials on volunteers from populations highly exposed to aflatoxins through their normal diet [242]. The levels of AFM1 biomarker in the urine of the volunteers treated with UPNS were reduced by 55% compared with placebo-treated controls. Unlike drug-based chemoprotection interventions, the inclusion of clay in the diet would be culturally acceptable in Africa and Asia where clay consumption was reported to be common in traditional ethnic groups of these regions [243]. In addition, studies demonstrated the absence of short-term adverse health effects in rats fed on rations containing up to 2% (w/w) of clay [244] as well as in poultry [245]. The high affinity of SN clays (montmorillonite and smectite) to aflatoxins suggests that they are unlikely to interfere with the absorption of micronutrients (e.g., metals and vitamins), as their action would be selectively directed towards aflatoxins [243]. Yet, further studies on long-term side-effects are needed to substantiate the safety, practicability, consumer acceptability, and risk-benefit profile of food-grade clays incorporation into the diet.

The enterosorption of aflatoxins in the GI tract to reduce their bioavailability can also be achieved by resident microbiota of the intestinal tract. Supply of probiotic bacteria with aflatoxin-binding properties to high-risk consumers in the form of encapsulated preparations or as biological supplements in fermented foods has also been recommended. Lactic acid bacteria commonly used in meat, vegetable, and dairy fermentations have been reported to bind and remove aflatoxins [246,247,248]. The “generally recognized as safe” status of these microorganisms and their worldwide use in food fermentations for millennia is an evident advantage with respect to their practicability and consumer-acceptability. Lactobacilli strains have received the most attention for their probiotic properties, including aflatoxin-bioavailability reduction, and for their reputed ability to colonize the intestinal tract. A particular focus was made on *Lb. rhamnosus* GC strain, which was demonstrated not only to bind and excrete aflatoxins in the feces causing the removal of up to 80%, but also to alleviate the associated liver injury and growth faltering in rats [247,249]. Other lactobacilli strains of different species grown under controlled conditions in the presence of AFB1 were demonstrated to bind and remove between 25 and 61% aflatoxin within 72 hours; *Lb. fermentum* and *Lb. plantarum* strains could remove instantaneously 56 and 61%, respectively [250]. Binding and removing AFB1 from a liquid solution at rates varying between 19 to 45% were recorded with *Enterococcus faecium* strains [251]. However, the results of these in vitro or animal studies are not directly transposable to humans with different metabolism, intestinal ecological conditions, and immunological response that may not be suitable for the candidate strains to grow and produce the anticipated effects within the GI tract. To simulate the human gastrointestinal conditions, an in vitro digestion model was designed to test lactobacilli and bifidobacterial strains for their ability to remove AFB1 from different food matrices, and the results showed moderate performances with a maximum reduction of 37% [252]. Given the ethical considerations that preclude administration of aflatoxins to humans in an experimental design, clinical trials on populations with high dietary exposure to aflatoxins, e.g., in Africa and Southeast China, remains the most appropriate alternative, but they are not always accessible and easy to perform. In addition, the results obtained on a given population may not apply to other populations in different parts of the world for genetic, ethnic, and culinary habit reasons. Nonetheless, studies on these approaches yielded inconsistent results regarding the percentage of aflatoxin removal, the time necessary for maximum binding, and parentage released back. In addition, the long-term effect on the equilibrium of the intestinal microbiota with possible adverse effects remain to be addressed [253].

Apart from the ability of resident intestinal microbiota to bind and remove aflatoxins, they can also interfere with their bioavailability by enzymatic degradation. Bacteria, such as *Escherichia coli, Bacillus* spp. *Pseudomonas* spp. *Stenotrophomonas* spp., *Arthrobacter* spp., and members of the family *Flavobacteriaceae* were shown in vitro to produce aflatoxin-degrading enzymes causing a significant reduction (>90%) in the levels of AFB1, AFM1 and/or AFB2 [254,255,256,257]. However, for these microorganisms to be effective in vivo, they have to colonize the intestinal tract and express sufficient levels of the aflatoxin-degrading enzymes. This may require specific physicochemical conditions and the presence of inducing factors for the enzyme to be optimally produced and active. A highly active aflatoxin-degrading enzyme produced by *Bacillus shackletonii* was indeed shown to require specific inducers for their expression and to be degraded by proteinase K which, in addition to the aerobic character of the producing bacterium, limit drastically its potential use as a probiotic strain in a chemoprotective strategy [255]. A suitable microorganism for a chemoprotection strategy should be able to adapt to the prevailing ecological conditions of the GI tract (*p*H, temperature, red/ox potential, and oxygen), to withstand the presence of inhibitory substances, e.g., bile and bile salts, and to compete with co-existing microorganisms, among other general requirements for a microbial strain to have the probiotic status [258]. These conditions narrow the scope of finding the ideal microbial strain where all the requirements are met, and no single strain, to our knowledge, was clinically studied for their effectiveness in degrading aflatoxins in humans. A suggested alternative was the use of multiple strains in one probiotic preparation [114,259], but maintaining the viability of the constitutive strains at appropriate ratios is the most challenging issue. A similar issue is experienced by cheese-making industry using complex starter cultures; the issue is certainly more problematic in the GI tract. Clinical trials were conducted on individuals from the population of Guangzhou, another southern Chinese region known for its high dietary intake of aflatoxins, to assess the efficacy of chemoprotection strategy using a combination (1:1) of two aflatoxin-binding probiotic bacteria (*Lb. rhamnosus* LC705 and *Propionibacterium freudenreichii* subsp. *shermanii*) [259]. The performance was assessed by monitoring aflatoxin-gua concentration in urine samples collected regularly during 5 weeks of treatment and 5 weeks after the treatment was ceased (a total of 10 weeks for the trail). A gradual decrease was observed in the mean urinary aflatoxin-gua concentration of the intervention group (receiving the probiotic capsules twice a day) compared with placebo group (receiving cellulose capsules) to reach a maximum of 55% reduction. Interestingly, no significant difference was observed in urinary concentration of the aflatoxin-gua biomarker between the two groups 5 weeks after the treatment termination, suggesting that the bacterial strains could not colonize the gut to become a part of its normal microbiota and sustain the modulatory effect on the toxicity of aflatoxins. 

A wide range of chemoprotection strategies based on the use of natural or synthetic drugs, foods and food constituents, and binders (microorganisms or inorganic non-nutritive materials) have been studied for their ability to provide some degree of protection against liver cancer caused by aflatoxins. However, none of them has been conclusively established as a universal application or shown to actually provide effective long-term chemoprotection for large-number populations. Plant-based chemoprotection appears to be promising, acceptable by consumers, and cost-effective, but there is still much to do in scientific research to soundly demonstrate its effectiveness, safety, and practicability. Combinations of different strategies or chemoprotective agents with different and complementary modes of action remain to be examined. Research in this field is seriously hampered by difficulties in performing clinical trials and the ethical prohibition of conducting experiments on human volunteers to whom aflatoxins should be administered. Most of the relevant clinical trials are conducted in only two Chinese regions, Qidong and Guangxi, where the dietary exposure to aflatoxins is unavoidably high, which may not be representative of the rest of the world. The development of reliable biomarkers for the prediction of aflatoxin-induced cancer early enough to allow testing safe and ethically acceptable strategies before the disease onset may help advance the scientific and applied research on chemoprevention. Moreover, the modern lifestyle with a clear trend of increased consumption of alcohol, tobacco, and fast and junk foods adds new challenges and risk factors that should be taken into consideration in scientific research on aflatoxins for future decades [1,149,260].

### 3.4. Legislative Challenges

The advent of globalization with the establishment of the WTO and the SPS agreement created the need to harmonize regulatory provisions for the flow of the global trade. Aflatoxins regulations have been one of the most challenging issues facing harmonization, as they are highly divergent throughout the world. While some countries set stringent standards to ensure the highest protection possible to public health, others have adopted more permissive standards aiming at an acceptable balance between “food safety” and “food availability” or to allow simplified trading with economic partners [261]. On the other hand, many countries have not promulgated yet aflatoxin regulations due to the associated high costs (food loss/condemnation, inspection, sampling, analyses, control at the borders, etc.) [179] and to insufficient institutional and capacity building for rigorous enforcement [261]. As a result, aflatoxins are inconsistently regulated across the globe regarding the aflatoxin types, food and feed commodities, and the standards in each item or group of items [261,262]. Depending on the country or region, the maximum tolerable limits (MTL) or the maximum limits (ML) can be set for AFB1 and/or for total aflatoxins (sum of AFB1, AFB1, AFG1, and AFG2); some countries set MLs for AFM1 in milk, dairy products, and/or infant formula. The MLs of the regulated aflatoxins are set for specific foods or foods in general, or for specific feeds or feeds in general, depending on the country or region (http://www.mycotoxins.info/en/regulations/, accessed on 7 July 2019). The general tendency of the standards is to be strict in industrialized countries and relaxed in developing countries, especially those of the tropics facing the highest problems to control aflatoxin contaminations. This lack of uniformity, added to the conflicting economic interest of each country, complicates greatly the harmonization process, especially when stringent regulations are imposed to developing countries or when scientific data to conduct meaningful risk assessment are missing or controversial. Nonetheless, harmonization has been fairly easy to reach in countries of the same geographical area and with comparable levels of development, such as the European Union (EU) countries, Australia and New Zealand, North America (USA and Canada), and South American countries members of the Southern common market (MERCOSUR), i.e., “Mercado Cómun del Sur”. At the global level, standards of each aflatoxin-commodity are matter of debate under the auspices of the *codex alimentarius commission* (CAC) before acceptance in the CA. The debate generally opposes industrialized, especially the EU, to developing countries. The first side claims strict standards to guarantee appropriate and due protection to consumers, and the second advocates relaxed standards to ensure fair trade and offer more economic opportunities to developing countries while maintaining an adequate level of safety [263]. It is generally admitted that imposing strict standards to developing countries affects severely health and economy, as they tend to export products of the highest quality and keep those of poor quality (high aflatoxin-contamination) for local consumption, which increases the exposure and hence health risk to local communities [263]. It has been argued, however, that strict standards with proper enforcement measures and international cooperation may benefit developing countries by forcing them to improve gradually the quality of their products and gain expertise through their interactions with industrialized countries, i.e., a “forcing technology” approach [264,265]. However, from the SPS agreement standpoint, unless strict standards are scientifically justified by health risk considerations, they would be regarded as prohibited technical barrier to trade. A study demonstrated that relaxing the ML of the total aflatoxins in tree nuts in the EU from 4 μg/kg to 10 μg/kg in 2009 benefited both EU-members and exporters from developing countries without undue increase in public health risks [263]. The two authoritative bodies for risk assessment studies, the FAO/WHO joint expert committee on food additives (JECFA) and the European food safety authority (EFSA), agreed that this shift would not adversely affect the public health. Conversely, a similar request to relax the UE standards of total aflatoxins in ready-to-eat (RTE) peanut from the current ML of 4 μg/kg to 10 μg/kg was refuted on the basis of EFSA risk assessment study suggesting that this change would cause an unacceptable additional increase of the risk by a factor of 1.6 to 1.8 [266]. The latter conclusion contrasts with the outcome of the JECFA assessment suggesting that an increase of ML from 4 μg/kg to 10 μg/kg will cause a marginal or no increase in the risk, but would reduce the rejection rate by more than 7% [267]. This reduction in the rejection rate was estimated to save about 233,333 metric tons of RTE peanut, corresponding to a trade value of about US$ 327 million [268]. The UE criticized the outcome of the latter study mainly from the standpoint of the sampling procedure and the regions of the world considered to determine the exposure. Similarly, a study conducted in Kenya demonstrated that the strict enforcement of the existing national standards for AFB1 in grains (maize, millet, and sorghum), and AFM1 in milk would deprive 9 million Kenyan consumers from their staples, and about 3.4 million consumers from milk; strict application of AFB1 standards in feeds was also suggested to result in a loss of 336,217 kg of milk [261]. Such a situation has been experienced in Serbia where setting the ML of AFM1 in milk to 0.05 μg/kg, for harmonization with the EU regulations, caused a major recall (62.3%) of the milk produced in 2013, urging the country to relax the standards back to 0.5 μg/kg next year [269].

Harmonization of aflatoxin regulations is mandatory to avoid litigious situations between member states of the WTO, but it will continue to raise challenges for at least the two next decades due to the highly divergent views on political economy, risk perception, and for scientific reasons. In addition to the lengthy procedure of the negotiations on a case-by-case basis within the Codex Committee on Contaminants in Foods (CCCF) and the Codex Alimentarius Commission (CAC). Countries with high-income and larger population tend to rise food safety issues and make lobbying efforts to impose standards that benefit their competitive economic sectors and provide them with tariffs and trade advantages; for a thorough discussion of this matter, see [270]. Use of science to shape regulatory provisions without bias is not always possible because of shortages in scientific criteria or data gaps to perform a sound quantitative risk assessment with undisputable conclusions. The current case of ML for aflatoxins in RTE peanut, discussed above, is one of the many cases that are being considered by the CCCF with difficulties to reach a consensus. Apart from the lack of reliable data of exposure in many parts of the world and for different groups of a population, sampling plans and analytical techniques to reach a risk assessment of a commodity-hazard combination have been criticized [264]. In addition, characterization of the risk related to aflatoxins has not been defined with certainty; the exact genotoxic dose of aflatoxins is not known and depends on various factors including the type of aflatoxin, individuals or group of individuals, and co-occurrence with other mycotoxins. It is being increasingly evident that co-occurrence of mycotoxins in foods and feeds is a very common phenomenon, and they interact with each other to produce toxicological effects that differ markedly in intensity and outcome from those produced individually [271]. This predicts the rise of new challenges related to the regulatory status of known or as yet to be discovered mycotoxins that may co-occur with aflatoxins possibly interfering with their toxicities and which may require to be regulated in their own right. 

Intensive scientific research is being conducted to address these challenges and provide new scientific information on aflatoxins to regulatory authorities. However, seeking the highest protection possible against aflatoxin intoxications by strengthening the standards may result in only a marginal reduction of the associated health risks while inducing excessive economic losses and food shortage with serious nutritional health consequences [263,271]. Although this is an international development issue which requires actions at different socio-economic and political levels, scientific research is a key instrument to define the borderline between objective science-based food safety regulatory provisions, on one hand, and socio-economic and international trade considerations, on the other hand. 

## 4. Conclusions

Since their discovery, aflatoxins have gained increased scientific interest due to their high impact on health, economy, and social life. During the sixties, scientific research on aflatoxins, which has been focused on their chemical characterization and toxicity-testing generated appreciable information of different types of aflatoxins and their association with liver cancer in various animals. This has initiated a large debate internationally about their hepatocarcinogenicity in humans and emphasized the need for their regulations in different commodities to secure food safety and ensure the flow of the trade. This debate has further stimulated research on aflatoxins aiming at improving the experimental design of clinical and epidemiological studies in order to yield sufficient and convincing evidence for aflatoxin carcinogenicity in humans. Biosynthesis and biodegradation pathways, and the mechanism of action were also intensively investigated to support the causal link between aflatoxins and liver cancer. Periodically, the IARC working group has performed critical reviews of the newly obtained results and appraised the advances made in the knowledge of these mycotoxins with regard to their health impact ultimately leading to the definite establishment of their carcinogenicity via genotoxic action. It was also established that the genotoxicity of aflatoxins is enhanced by chronic HB virus infection, although the mechanism of this synergy remains to be demonstrated. 

In the meantime, sustained efforts have been deployed worldwide to limit the health risks associated with aflatoxins by reducing the exposure via dietary intake. The use of high-tech agricultural management system allowed industrialized countries to tackle efficiently the problem and provide a reasonable protection to their consumers, which has encouraged them to promulgate increasingly stringent standards. In contrast, most developing countries, especially the tropical and sub-tropical continue to struggle with the high incidence of aflatoxin contamination of foods and feeds due to their economic vulnerability and the favorable climatic conditions for aflatoxigenic mould growth and aflatoxin production. Therefore, most of these counties are unable to adopt and enforce strict standards, which limits their access to the markets of industrialized countries and creates an imbalance in agri-food international trade. This issue also continues to be a matter of debate within the WTO to avoid disguised use of food safety regulations as a technical barrier to fair trade. The SPS agreement emphasizes the need for science-based regulatory provisions standards using risk assessment as the scientific basis to determine the standards to protect human and animal health while facilitating global trade of agri-foods. However, food safety risk assessment is a relatively recent discipline and does not provide indisputable answers to all issues raised due to the lack of the necessary data, e.g., exposure and/or the exact toxic dose for many hazards, including aflatoxins. This debate will continue to challenge the international community for decades to come awaiting the generation of meaningful risk assessments of aflatoxins as standalone hazards and/or as part of multi-mycotoxin contaminant in foods and feeds. 

Despite the scientific progress in the knowledge on aflatoxins and the efforts made to reduce the risk they pose to public health, developing countries still have to tolerate high level of contamination of foods and feeds to not compromise food supply. Therefore, alternative ways to mitigate health risk by detoxifying already contaminated foods and feeds before consumption or by acting on consumers to interfere with aflatoxin toxicity or bioavailability in situ have been suggested. All proposed interventions have yielded inconsistent results to provide definite and practicable solutions at large scale, and further refinements are needed before their efficacies and conditions of their use are recognized. The impact of the presently suggested intervention approaches will be complicated in the future by the climatic change with a clear trend to enhance the risk of aflatoxins as well as the change in the lifestyle that introduces additional risk factors. In fact, it is evident that reducing aflatoxin contamination and associated health risks cannot be achieved by specific interventions, but rather by concerted actions at many levels and by all stakeholders. These actions should include education, scientific research, legislation framework, environment protection to limit climatic change, and international cooperation for sustainable development to fight hunger and poverty. Partnership and research programs on aflatoxins should be reshaped to be oriented towards practicable solutions with greater involvement of social scientists. The formation of research and association networks between developing and industrialized countries would play a paramount role in creating a synergy to ensure information exchange and technology transfer in order to bridge the gap between the North and South hemispheres of the globe.

## Figures and Tables

**Table 1:** Main physicochemical properties of aflatoxins as originally determined by Hartley, Nesbitt and O’Kelly [33].

Aflatoxin	Crystallization Solvents	Fluorescence under UV Light (360 nm)	Fluorescence Spectrum		MW	Melting Point °C	Chemical Formula
			Excitation Wavelength (nm)	Emission Wavelength (nm)			
Aflatoxin B1	Trichloroethylene/chloroform	Blue	365	425	312	265–270	C_17_H_12_O_6_
Aflatoxin B2	Methanol	Blue	365	425	314	305–309	C_17_H_14_O_6_
Aflatoxin G1	Methanol	Green	365	450	328	247–250	C_17_H_12_O_7_
Aflatoxin G2	Ethanol	Green-Blue	365	450	330	237–240	C_17_H_14_O_7_

**Table 2:** Major milestones in the historical and scientific progress leading to the contemporary knowledge on aflatoxins (up-to 2012).

Year	Study	Action/Outcome	References
1960	Outbreak of turkey “X” disease in the Eastern and Southern regions of London (England) poultry farms	Starting a wide investigation to understand the disease	[2]
1961	Association of the X disease to the imported Brazilian groundnut meal	Removal of Brazilian groundnut from rations and reduction of the disease incidence	[2]
	In vivo confirmation of the toxicity of Brazilian groundnut to other birds and animals	Characterization of typical symptoms and organ lesions caused by the disease	[2,18,19,20,21,22,23]
	Preparation of a concentrate of the toxic principle in an aqueous suspension	Development of reliable quantitative biological toxicity assay	[22]
	Association of *Aspergillus flavus* contamination with the toxicity of groundnut	Establishing the relation between toxic fractions from cultures of *A. flavus* and fluorescence under UV light	[18]
1962	Improving separation and purification techniques of *A. flavus* culture extracts by thin-layer chromatography (TLC)	New separation methods for aflatoxin purification Identification of aflatoxins B and G	[30]
	Developing crystallization methods Studies on physicochemical properties of aflatoxins	Preparation of crystalline aflatoxins Isolation and chemical characterization of aflatoxin B1	[29,31,32]
1962	Animal trials for the fate of aflatoxin after ingestion of contaminated feed	Detection of a toxic principle, “milk toxin”, in milk drawn from cows fed on toxic groundnut	[36]
1963	Purification and crystallization of aflatoxins	Identification and chemical characterization of aflatoxins B1, B2, G1 and G2	[33]
		First chemical synthesis of aflatoxins G1 and G2	
	Structural studies of aflatoxins B and G	Elucidation of the chemical structures of aflatoxins B and G as difuranocoumarin derivatives	[34,35]
1964	Purification and toxicity testing of the “milk toxin” in dry milk	Partial characterization of the “milk toxin” and its relatedness to aflatoxin B1	[40]
1965	Setting safety standard by the United States Food and Drug Administration (US FDA)	First regulation of total aflatoxins (ML of 30 *pp*b in foods) in the USA.	[60]
1966	Sheep fed on a mixture of aflatoxins to determine organs and/or body fluids secretions where the “milk toxin” is accumulated or secreted	Detection of “milk toxin” in the milk, urine, kidney, and liver Designation of the “milk toxin” as “aflatoxin M”	[38]
	Purification and fractionation of Aflatoxin M from sheep urine	Separation and physicochemical characterization of aflatoxins M1 and M2 as hydroxylated metabolites of aflatoxins B1 and B2, respectively	[39]
	Feeding malnourished African children with meals supplemented with peanut as part of an initiative of the United Nations Food and Agricultural Organization (FAO) to control kwashiorkor	Liver damage in most children having been beneficiary participants in the initiative	[51]
1967	Studies on in vitro chemical characterization of aflatoxins	Complete chemical synthesis of aflatoxin B1	[82]
1969	Revising standard levels by the US FDA	Action level for total aflatoxins in foods lowered from 30 *pp*b to 20 *pp*b	[60]
1970	A case-control study on liver failure leading to the death of a teenager fed on mouldy cassava in Uganda	Circumstantial evidence of the implication of aflatoxins in acute intoxication	[54]
1971	First review by the International Agency for Research on Cancer (IARC) working group of the available studies on the possible relationship between aflatoxin intake and liver cancer	Studies reviewed were considered to provide a circumstantial evidence for the carcinogenicity of aflatoxins in humans	[56]
1975	Second review by the IARC working group of the previous and newly generated data on the causality between aflatoxins and liver cancer	Confirmation of the previous status of “circumstantial evidence” for carcinogenicity in humans	[61]
1977	Regulatory action guidelines of the US FDA	Action level of 0.5 *pp*b for aflatoxin M1 in milk	[83]
1979	Building evidence for a link between liver damage and aflatoxin intake	The FAO establishes the first provisional acceptable limit of 30 mg aflatoxin per kg meal	[53]
1987	Third review of new available data generated from better-designed studies to address previous recommendations of the IARC working group	Classification of naturally occurring mixtures of aflatoxins in group 1 carcinogens	[62]
1991	Development of mechanistic studies to demonstrate the carcinogenicity of aflatoxins at the molecular level	Demonstration of the genotoxicity of aflatoxin by induction of point mutation in codon 249 of *TP53* tumor suppressor gene	[71]
1992	High-quality design of epidemiological and mechanistic studies on the carcinogenicity of aflatoxins	Establishment of an almost linear relationship between AFB1 intake and liver cancer Demonstration of synergistic action between dietary intake of aflatoxins and hepatitis virus B hepatocellular carcinoma First use of Aflatoxin P1, Aflatoxin M1, and DNA-adduct in urine as a biomarker for the exposure assessment	[63]
	Review of the newly generated data by the IARC working group	Addition of Aflatoxin B1 to the group 1 carcinogens	[66]
1997	Cohort studies taking into account available biomarkers to confirm the carcinogenicity of Aflatoxin B1 by	Recommendation of wider use of biomarkers as reliable tools to assess exposure to aflatoxins and for aflatoxicosis diagnostic	[64,65]
2002	Review of new and previous data on the carcinogenicity of aflatoxins by the IARC working group	Confirmation of the previous status of aflatoxins	[67]
2004	Investigations on a large aflatoxicosis that occurred in Kenya	The first use of aflatoxin-albumin adduct in blood serum as a biomarker for aflatoxin exposure	[70]
2012	Review of previous and new data on aflatoxin carcinogenicity with an emphasis on mechanistic studies on the genotoxicity of aflatoxins and biomarkers	Revision of the previous classification to consider aflatoxins, implicitly including Aflatoxins B1, B2, G1, G2, and M1 in group 1 carcinogens *	[74]

* In 2012, the IARC classified aflatoxins in group 1 carcinogens without specifying “mixtures of naturally occurring aflatoxins” and aflatoxin B1 as in the previous versions of the IARC monographs, thereby implicitly including the major aflatoxins (B1, G1, B2, G2, and M1) in this group on the basis of strong evidence for their genotoxicity involving the formation of DNA adducts causing point mutations in the *TP53* gene [74].

**Table 3:** Biomarkers used to assess exposure or risk posed by aflatoxin B1 in humans, limitations and strengths.

Biomarker	Limitations	Strengths	Detection Specimen	Validation Status	References
Aflatoxin B1 (parent) *	No correlation with the ingested amount of the aflatoxin	Useful when used along with other biomarkers	Urine and serum	No	[65,133,134]
**Phase I Metabolites**					
Aflatoxicol *	Lack of correlation with aflatoxin intake	May be useful when used along with other biomarkers	Urine	No	[135]
Aflatoxin M1 *	Significance for short term exposure only	Major aflatoxin B1 metabolite excreted in the urine. Highly correlated with aflatoxin B1 dietary intake. Evidence for a dose-response relationship with hepatocellular carcinoma (HCC)	Urine	Yes	[65,122,123]
Aflatoxin P1 *	Significance for short term exposure only. Lack of correlation with aflatoxin B1 intake	Useful when associated with other biomarkers in providing information on the risk of disease onset and diagnosis	Urine	No	[63,65,117]
Aflatoxin Q1 *	Significance short term exposure only. Rarely detected in the urine after exposure to aflatoxin B1	May be useful if used along with other biomarkers	Urine	No	[65,136,137]
Aflatoxin B2 _a_ *	Does not necessarily reflect the DNA damaging effect of aflatoxin B1. Lack of correlation with aflatoxin B1 intake	One of the major metabolites of aflatoxin B1 which may inform on acute toxicity (forms adducts with proteins and phospholipids)	Serum, Urine	No	[116,138]
Aflatoxin B1-8,9-dihydro-diol **	Not excreted in biofluids; no easily accessible or available samples	May be a good indicator for acute toxicity, as it leads to the formation of adducts with functional proteins	Liver (in vitro)	No	[117]
Aflatoxin B1-exo-8,9-epoxide **	Not excreted in biofluids; no easily accessible or available samples. Short-lived, very unstable intermediate metabolite (difficult to quantitate accurately any time after ingestion)	Directly related to toxicity mechanism; best risk marker for the aflatoxin intake, dose/response determinations, and prediction of the disease onset	Liver (in vitro)	No	[72,139,140]
**Phase II Metabolites (Aflatoxin-Protein Adducts)**					
Aflatoxin B1-lysine **	Limited value regarding quantitative risk assessment and the prediction of HCC at early stages	Chronic exposure (stable for more than 3 months in serum). Highly correlated with aflatoxin B1 dietary intake Best used to associate aflatoxin intake and child growth impairment	Blood serum	Yes	[84,113,129,131,141,142]
**Phase II Metabolites (Aflatoxin DNA Adducts)**					
Aflatoxin B1-N^7^-Guanine **	Not valid for a long-term exposure or to predict the onset of liver cancer	Linear proportionality with ingested aflatoxin B1. Confirmation of the etiology of aflatoxins in HCC	Urine	Yes	[122,130]
Aflatoxin B1-FAPY **	Not excreted in biofluids; no easily accessible or available samples	Directly implicated in DNA mutations leading to cancer; suitable marker for dose-response determination and disease outcome	Liver, kidney, viscera (in vitro)	No	[119]
Aflatoxin B1-8,9-dihydro-diol **	Idem as aflatoxin B1-FAPY	May be a good indicator for acute toxicity, as it leads to the formation of adducts with functional proteins	Liver (in vitro)	No	[117]
Aflatoxin B-exo-8,9-epoxide **	Idem as aflatoxinB1-FAPY Short-lived, very unstable intermediate metabolite	Directly related to toxicity mechanism; best risk marker for the aflatoxin intake, dose/response determinations, and prediction of the disease onset	Liver (in vitro)	No	[72,139,140]
Aflatoxin-glutathione	Idem as aflatoxin B1-FAPY. Unavailability for sampling; only detected in bile	Best indicator for the detoxification of aflatoxins	Bile	No	[143]
Aflatoxin-mercapturic acid *	Low level in urine does not necessarily indicate a shift towards the genotoxic pathway if the phase I enzymes are inhibited (low levels of epoxides)	Good risk biomarker to monitor the outcome of chemoprotective actions; reflects the induction of the detoxification pathway via aflatoxin B1–GST conjugate formation (phase II metabolites activation)	Urine	No	[120,125,144]
Aflatoxin-glucuronide	Insufficiently studied	Indicator of the of aflatoxin aldehyde reductase (AFAR) activity; potential use to monitor chemoprotection action by administering AFAR inducers	Urine	No	[80]

* Biomarkers for determinations of the “internal dose”, as a measure of the products that are produced in the body from the metabolism of the parent aflatoxin to serve as exposure biomarkers. ** Biomarkers for determinations of the “biologically effective dose”, i.e., “the fraction of xenobiotic capable of interacting with cellular macromolecules at the target site. Macromolecular adducts should not only be considered as exposure indicators; indeed, their biological significance can also be extended to biomarkers of effect and of susceptibility” [145].
